# SARM1 Promotes Neurodegeneration and Memory Impairment in Mouse Models of Alzheimer's Disease

**DOI:** 10.14336/AD.2023.0516-1

**Published:** 2024-02-01

**Authors:** Xuemeng Miao, Qian Wu, Siyu Du, Ludan Xiang, Siyao Zhou, Junzhe Zhu, Zirun Chen, Hui Wang, Xuyi Pan, Yiren Fan, Lihan Zhang, Jingkang Qian, Yuxuan Xing, Yiyang Xie, Lixin Hu, Haiyun Xu, Wei Wang, Ying Wang, Zhihui Huang

**Affiliations:** ^1^College of Pharmacy, Hangzhou Normal University, Hangzhou, Zhejiang 311121, China.; ^2^School of Mental Health, Wenzhou Medical University, Wenzhou, Zhejiang 325035, China.; ^3^School of the First Clinical Medical Sciences, School of Information and Engineering, Wenzhou Medical University, Wenzhou, Zhejiang 325205, China.; ^4^Clinical Research Center, Affiliated Hangzhou First People’s Hospital, Zhejiang University School of Medicine, Hangzhou, Zhejiang 310003, China.

**Keywords:** Alzheimer’s disease;, SARM1, degeneration, inflammation, memory

## Abstract

Neuroinflammation plays a crucial role in the pathogenesis and progression of Alzheimer's disease (AD). The Sterile Alpha and Toll Interleukin Receptor Motif-containing protein 1 (SARM1) has been shown to promote axonal degeneration and is involved in neuroinflammation. However, the role of SARM1 in AD remains unclear. In this study, we found that SARM1 was reduced in hippocampal neurons of AD model mice. Interestingly, conditional knockout (CKO) of SARM1 in the central nervous system (CNS, SARM1^Nestin^-CKO mice) delayed the cognitive decline in APP/PS1 AD model mice. Furthermore, SARM1 deletion reduced the Aβ deposition and inflammatory infiltration in the hippocampus and inhibited neurodegeneration in APP/PS1 AD model mice. Further investigation into the underlying mechanisms revealed that the signaling of tumor necrosis factor-α (TNF-α) was downregulated in the hippocampus tissues of APP/PS1;SARM1^Nestin^-CKO mice, thereby alleviating the cognitive decline, Aβ deposition and inflammatory infiltration. These findings identify unrecognized functions of SARM1 in promoting AD and reveal the SARM1-TNF-α pathway in AD model mice.

## INTRODUCTION

Alzheimer's disease (AD) is a chronic, degenerative neurological disorder that accounts for 60-80% of dementia case [1]. It affects more than 50 million people globally, and its incidence increases with age, from 5% to 10% at age 65-75 to around 15% at age 85, and over 50% at age 90 and older [2]. As the leading cause of senile dementia, AD is a progressive and neurodegenerative disease that primarily affects the elderly, characterized by symptoms such as memory loss, cognitive impairment and behavioral abnormalities [3]. Memory loss is the most prominent and severe symptom throughout the course of AD [4]. The high prevalence and progressive nature of AD results in significant economic burden and psychological distress for affected individuals and their families.

The main pathological features of AD are the deposition of amyloid beta (Aβ), neuronal fiber tangles, and neuronal loss [5]. Among these, the accumulation of toxic Aβ peptides leads to synaptic dysfunction, axonal degeneration, and eventually neuronal death [6]. Recent studies have shown that increased inflammation raises the risk of developing AD [7], while long-term suppression of inflammation reduces its onset and may delay its progression [8]. Although the inflammatory response is thought to have a beneficial role in inhibiting the early stages of disease progression, chronic neuroinflammation and associated pro-inflammatory cytokines can eventually lead to neuronal death [9]. Aβ can activate microglia and astrocytes, which subsequently secrete a large number of inflammatory factors such as TNF-α and IFN-γ and produce neurotoxic substances that can cause neuronal dysfunction [10]. A significant increase in NF-κB phosphorylation was observed in APP/PS1 AD model mice, suggesting that Aβ can also activate NF-κB [11]. Importantly, the accumulation of Aβ and the accompanying inflammatory response ultimately lead to neuronal death and cognitive decline [12]. Therefore, neuronal dysfunction and neuroinflammation may play crucial roles in the pathogenesis and progression of AD. However, the underlying mechanisms and consequences for the pathogenesis and other conditions of AD remain elusive.

Toll-like receptors (TLRs) are key components of the innate immune system and are expressed in the CNS of mammals. They are widely distributed in various regions of the brain, including the hippocampus, amygdala, cerebral cortex, midbrain, and cerebellum and their expression in neurons is higher than in astrocytes and microglia [13]. SARM1 (sterile alpha and TIR motif containing 1) is an adaptor protein for the TIR domain of the downstream signal of TLR, which has the Toll/interleukin-1 receptor domain [14]. *In vitro* studies have shown that SARM1 mediates neuronal morphological changes by regulating dendritic structure, axonal growth, and neuronal polarity in hippocampal neurons [15]. Additionally, SARM1 promotes Wallerian degeneration induced axonal death [16]. More importantly, SARM1 plays a pivotal role in the neuroimmune response. In SARM1 knockdown mice, the expression of cytokines such as IL-6 and IFN-β are altered in the brain, indicating that SARM1 is fundamental in the neuroimmune response [17]. Young SARM1 knockout mice do not have any obvious phenotype and furthermore display normal exercise performance, but the loss of SARM1 prevents neuron degeneration in models of spinal cord injury, traumatic brain injury, peripheral neuropathy, and retinal degenerative diseases [18-21]. However, the role of SARM1 in AD remains unclear.

In the present study, we examined the functions of SARM1 in the CNS on cognitive function and associated pathophysiological changes in AD model mice based on APP/PS1;SARM1^Nestin^-CKO mice. Our results showed that the deletion of SARM1 in APP/PS1 AD model mice led to a reduction in Aβ deposition, inflammatory infiltration, and neuronal death, thereby delaying cognitive decline. Mechanistically, our findings suggest that SARM1 promoted AD progression through the TNF-α pathway. Our study sheds light on the underlying mechanisms of AD, and provides potential targets for the treatment of AD.

## MATERIALS AND METHODS

### Mouse breeding and genotyping

SARM1^f/f^ mice were crossed with Nestin-Cre transgenic mice (003771, from The Jackson Laboratory) to generate SARM1^Nestin^-CKO mice. SARM1^f/f^ mice and Nestin-Cre transgenic mice were maintained in the C57BL/6J strain background. These SARM1^Nestin^-CKO mice were subsequently bred with APP/PS1 double transgenic mice (obtained from Southern Model Animal Center, Nanjing) to produce the experimental group of APP/ PS1;SARM1^Nestin^-CKO mice. The control group consisted of SARM1^f/f^, SARM1^Nestin^-CKO, and APP/ PS1;SARM1^f/f^ mice. No significant differences in livability or body weight were observed among the four groups. All mice were subjected to a 12 h light/dark cycle and raised according to standard scientific procedures.

A polymerase chain reaction (PCR) was performed to genotype the mice using specific primers. The primer sequences used were as follows: for APP/PS1, 5'-GACTGACCACTCGACCAGCTT-3' and 5'-CTTGTA AGTTGGATTCTCATAT-3 [22]; for SARM1 forward 5'-AGCAACAAGCACTCTGAATGG-3 and reverse 5'-AGATCACGCCTAGACCGATG-3'. Only male mice were selected for this study to reduce the potential bias caused by gender differences in behavioral testing. At the end of the behavioral tests, the mice were anesthetized, perfused with normal saline, and fixed with paraformaldehyde (PFA).

All animal studies involving transgenic and knockout mice were approved and conducted in accordance with the guidelines and regulations set by the Animal Care and Use Committee of Wenzhou Medical University (IACUC no: 2018-666242).

### Mouse behavior tests

All behavioral analyses were conducted on adult mice, and experimental tests were scheduled within fixed time periods to minimize experimental variations. To maintain consistency in the experimental procedure, the behavioral tests were performed in a specific sequence. For SARM1^f/f^ and SARM1^Nestin^-CKO mice, the behavioral assays were conducted in the following order: sucrose preference test, open field test, Y-maze test, novel object recognition test, Barnes maze test, elevated plus maze, Morris water maze, tail suspension test and forced swimming test. The same order of behavioral assays was followed for the APP/PS1;SARM1^f/f^ mice and APP/ PS1;SARM1^Nestin^-CKO mice. Additionally, the human observers were blinded to the group allocation.

#### Sucrose preference test

The sucrose preference test (SPT) was widely used to detect the symptoms of depression [23, 24]. Briefly, each mouse was individually placed in a specific size cage, given sufficient food and two bottles, one of which had a 1% sucrose solution while the other bottle contained water. During the experiment, the positions of the two bottles were exchanged every day to avoid the influence of position preference. Sucrose intake was an indicator of reward sensitivity in mice. The SPT was divided into two phases. The first phase was the three-day adaptation period for mice, and the second phase was the fasting and water deprivation phase. After 24 h of water and food deprivation, the formal process of weighing started. Mice were measured for sucrose and water intake at 24 h, 48 h, and 72 h. The measure of sucrose preference was calculated as follows: Sucrose preferences (%) = sucrose consumption / (sucrose + water consumption) × 100%.

#### Open field test

To assess locomotion, anxiety, and stereotypical behavior states in mice, we conducted the open field test (OFT) which reflects spontaneous activity levels [25]. The experimental site was kept quiet, and lighting was appropriate. The mice were placed in the center of an uncovered acrylonitrile butadiene styrene box (40 × 40 cm) and allowed to explore freely for 10 min. Ethovision XT 13 software (Noldus) was used to record the total distance and time spent exploring the central (middle 20 × 20 cm size) and peripheral areas. After each experiment, the box was scrubbed with 75% ethanol to minimize disturbance to the mice.

#### Y-maze test

A Y-maze consisting of three identical arms was used to further examine spatial learning and memory [26]. The mice were placed at the end of one arm and allowed to explore the maze freely for 10 min. The time and frequency of entering each arm and the sequence of arm entries were recorded. The surface of the Y-maze was cleaned with 75% ethanol after each test to remove any odor cues.

#### Novel Object Recognition test

The novel object recognition test (NRT) was designed to assess the cognitive ability of mice based on their exploratory instincts [27]. During the first phase, the mice were allowed to orient themselves in the environment for 30 min before exploring a nontransparent plastic box for 5 min. The habituation phase comprised spending 15 min in the same box on both day 2 and day 3. On the next phase, two identical objects were placed in opposite positions in the box, and the mice were allowed to explore them for 10 min. After a one-hour interval, the mice were returned to the box, and one of the familiar objects was replaced with a new novel object. This phase lasted for 10 min and the box and objects were cleaned with 75% ethanol after each session. Only when the mice were touching the object with their nose and/or directing their nose to the object within 2 cm, was the exploration time of each object recorded. The preference scores were calculated as the time spent exploring new objects divided by the total time spent exploring familiar and new objects.

#### Barnes maze test

The Barnes maze test consisted of a white round table and a black escape box [28]. The tabletop was 92 cm in diameter and had 20 holes equally spaced around the center, with a target hole and a black plastic escape box (15 × 7 × 7 cm) below it. The mice were placed in a stainless-steel tank with a diameter of 120 cm and a height of 50 cm. The environment was designed to include stimuli such as strong wind and light, to help the mice quickly find and hide in the target hole. The first day of the experiment was the adaptation period, during which each mouse was guided into the hole twice and stayed in the hole for 3 min. The experiment interval for the same mouse was at least 10 min. The experiment period lasted from day 2 to day 6. The difference from the previous day was that each experiment was performed 3 times per day at 15 min intervals. In each experiment, the mice were placed in a dark box in the center of the maze for 10 s, and then they had to find the target hole within 4 min, otherwise, they were guided into the hole and stayed for 1 min. The platform and escape box were thoroughly cleaned up with 75% ethanol and paper towels between each experiment to eliminate the effect of odor between mice.

#### Elevated plus maze

The elevated plus maze (EPM) was a commonly used test to assess anxiety in mice, and it was performed under quiet and appropriately lit conditions [29]. The maze was about 40 cm high and consisted of two open arms (30 × 5 × 0.5 cm) and two closed arms (30 × 5 × 15 cm). The mice were placed in the central area facing the open arms and allowed to explore the maze freely for 5 min. The frequency and time of mice exploring the open and closed arms were recorded using Ethovision XT 13 software (Noldus). After each experiment, the maze was wiped with 75% ethanol to minimize interference to the mice.

#### Morris Water Maze

The Morris Water Maze (MWM) was used to test spatial memory in mice [30, 31]. In short, the circular MWM apparatus was filled with water and contained an immersion escape platform (10 cm in diameter) located 1 cm below the water surface. The MWM was sprinkled with white powder to make the water milky white, divided into four quadrants, and the water temperature was kept at 24 ± 1°C.

The mice were tested five times a day (swimming time 60 s, test interval 60 min) for 5 d, with each swimming session lasting for 60 s and a 60 min interval between each test. On the second day after the last training, the platform was removed, and the percentage of time the mice spend in the quadrant where the platform had been located, as well as the number of times it entered that quadrant, were recorded as indicators of spatial memory capacity. It should be noted that the water in the pool was replaced regularly to maintain optimal experimental conditions.

#### Tail suspension test

The tail suspension test (TST) was a classic experiment that evaluated the symptoms of depression [32]. The mice were taped to the wire, placed about 1.5 cm from the tail and hung 30 cm above the floor. The times of struggling and immobility were recorded during the experiment. In short, the tail of the mouse was fixed on a hanging hook with tape to make mice completely suspend in an inverted state. The whole experiment lasted 6 min, with the first minute set as the adaptation time and the last 5 min as the recording time.

#### Forced swimming test

The basic principle of the forced swimming test (FST) was that when mice were placed in a 2 L glass beaker with 1.6 L of water, the water temperature was controlled at 24°C, and the mice were placed in the beaker to swim for 6 min [33]. Initially, the mice tried desperately to swim to escape, but soon they became floating and immobile, and their limbs occasionally paddled to keep their bodies from sinking, which actually meant that the animals had given up the hope of escape and had sunk into behavioral despair. The immobility time of the mice was recorded during the last 4 min of the 6 min experiment.

### Nissl staining

Nissl staining was carried out following the protocol described [34]. Briefly, the mice were first treated with 0.1 mol/L PBS and then perfused with 4% PFA. They were then fixed by soaking in 4% PFA for 24 h and subsequently dehydrated in 30% sucrose before being cut into 20 μm thick longitudinal sections using a frozen microtome (Thermo, US). The frozen sections were then incubated with 0.1% cresol purple at room temperature for 6 min, followed by rinsing with double distilled water and 95% ethanol, and then dehydration with 100% ethanol and transparency with xylene. A neutral resin was used to mount the coverslip. The images were obtained using a digital slice scanner (SLIDEVIEW VS200, Olympus Corporation, JPN) and were quantitatively analyzed using Image J.

### Immunostaining

The mice were perfused with 0.1 mol/L PBS, and then brain tissues were immersed in 4% PFA for 24 h and transferred to a 30% sucrose solution for 3 d for dehydration. Subsequently, the brain tissues were cut into 20 μm thick longitudinal sections using a frozen microtome (Thermo, USA). For staining the brain tissue sections, the sections were fixed with PFA for 30 min and then repaired for 30 min with sodium citrate antigen repair solution (Solarbio) in a water bath at 90°C. Non-specific antigens in the brain tissue sections were blocked with 5% BSA plus 0.3% Triton X-100 for 1 h at room temperature. Next, they were incubated with the primary antibody overnight at 4°C and then washed three times with PBS. A suitable secondary antibody (1:1, 000, Invitrogen) was selected and incubated at room temperature for 1 h. The main primary antibodies used included rabbit anti-SARM1 (ab226930, Abcam, 1:200), mouse anti-NeuN (MAB377, Millipore, 1:200), rabbit anti-NeuN (ab177487, Abcam, 1:400), mouse anti-GFAP (MAB360, Millipore, 1:500), rabbit anti-GFAP (ab7260, Abcam, 1:500), goat anti-Iba1 (ab5076, Abcam, 1:500), rabbit anti-Iba1 (ab178846, Abcam, 1:500), rabbit anti-CD45 (ab10558, Abcam, 1:500) and mouse anti-Aβ (Covance 6E10, BioLegend, 1:500). Sections were stained with DAPI (1:1, 000, Sigma-Aldrich) to show the nucleus. For thioflavin-s staining, brain sections were incubated with 0.015% thioflavin-S (T1892; Sigma Mo, USA) reagent for 15 min at room temperature [35]. Each experiment labeled and analyzed sections derived from both control and experimental groups to ensure internal experimental consistency. All images for a given immunostain were taken using the same exposure time, deconvolved using equivalent settings. Images were acquired using Delta Vision Ultra (GE) and a confocal microscope (Zeiss). Image J and Photoshop (Adobe) software were mainly used for analysis. There was no positive signal observed in the control incubations without primary antibody.

### Quantitative Real-Time PCR (qRT-PCR)

For qRT-PCR, total RNA was extracted from the hippocampus of approximately 9-month-old SARM1^f/f^, SARM1^Nestin^-CKO, APP/PS1;SARM1^f/f^ and APP/ PS1;SARM1^Nestin^-CKO mice at using TRIzol^TM^ Reagent (#15596026, Ambion), following the manufacturer’s instructions. The RNA was then converted into cDNA using the SuperScript^TM^ One-Step Reverse Transcription Kit (#10928-034, Invitrogen, CA, USA). RT-PCR detection system (Applied Biosystems, USA) was used to measure the expression levels of mRNA, and the iTaq^TM^ Universal SYBR Green Supermix (Bio-Rad, USA) was used. Samples were independently amplified at least three times. The 2-ΔΔCt method was used to convert the relative gene expression against β-actin. The β-actin primer sequences were as follows: forward, 5'-AAGGAAGGCTGGAAAAGAGC-3' and reverse, 5'-GCTACAGCTTCACCACCACA-3'. TNF-α primer sequences were as follows: forward, 5'-ACGTGGAACT GGCAGAAGAG-3' and reverse, 5'-CTCCTCCACTT GGTGGTTTG-3' [36].

### Western blotting

Western blotting was conducted following the previously described method [37]. The hippocampus tissues were homogenized and lysed in a lysis buffer consisting of 50 mM Tris-HCl (pH7.4), 150 mM NaCl, 1% NP-40, 0.5% Triton X-100, 1 mM phenylmethylsulfonyl fluoride (PMSF), 1 mM EDTA, 5 mM sodium fluoride, 2 mM sodium orthovanadate and protease inhibitor cocktail (Sigma, P8340) at 4°C for 30 min. The lysates were then centrifuged at 12, 000 rpm for 30 min and the supernatants were collected. The proteins were extracted from the supernatants using 5× loading buffers and boiled at 100°C for 10 min. The protein samples were separated using sodium dodecyl sulfate-polyacrylamide gel electrophoresis (SDS-PAGE) with an 8%, 10%, or 12% and transferred onto a nitrocellulose membrane (Life sciences, USA). The membrane was blocked in 5% skimmed milk at room temperature for 1 h and then incubated with primary antibodies, including rabbit anti-SARM1 (ab226930, Abcam, 1:1000), rabbit anti-NF-κB p65 (WL01980, Wanleibio, 1:500), rabbit anti-p-JNK (ET1609-42, HuaBio, 1:500) and rabbit anti-TNF-α (R1203-1, HuaBio, 1:1, 000) overnight at 4°C. Mouse anti-β-actin (A5316, Sigma-Aldrich, WB 1:10,000) was used as the loading control. After washing the membrane three times with TBST, it was incubated with horseradish peroxidase (HRP)-conjugated secondary antibodies for 1 h. The protein signals were detected using the ECL kit (Bio-Rad, USA). Finally, immunoblots were analyzed using the quantity-one software (Bio-Rad, USA).

### Drug injection

The 6-month-old APP/PS1;SARM1^f/f^ and APP/ PS1;SARM1^Nestin^-CKO mice were treated with R-7050 (6 mg/kg i.p., GLPBIO, catalog # GC11659) twice weekly for 3 months until the experiments were terminated [38, 39]. As a control group, another batch of APP/ PS1; SARM1^f/f^ and APP/PS1;SARM1^Nestin^-CKO mice were intraperitoneally injected with the same volume of saline.

### Statistics and reproducibility

All data presented in this study are expressed as arithmetic mean ± SEM. Statistical analyses were conducted using GraphPad Prism version 8.0, with null hypotheses being rejected at *P ≥ 0.05*. Prior to conducting statistical comparisons between two groups, we first performed a Shapiro-Wilk normality test (Prism) to examine normal distribution of the data. One-way ANOVA was utilized to determine statistically significant differences between groups. In cases of multiple comparisons, Bonferroni correction (≤ 6 groups) or Tukey's correction (> 6 groups) was employed to adjust P values accordingly, thereby lowering the probability of type I errors.

To ensure reproducibility, all experiments conducted in this study involved a minimum of three mice or independent experiments. Moreover, all studies conducted on mice were repeated at least once to validate the consistency of results.

## RESULTS

### SARM1 expression was reduced in hippocampal neurons of AD model mice

**Figure 1.**
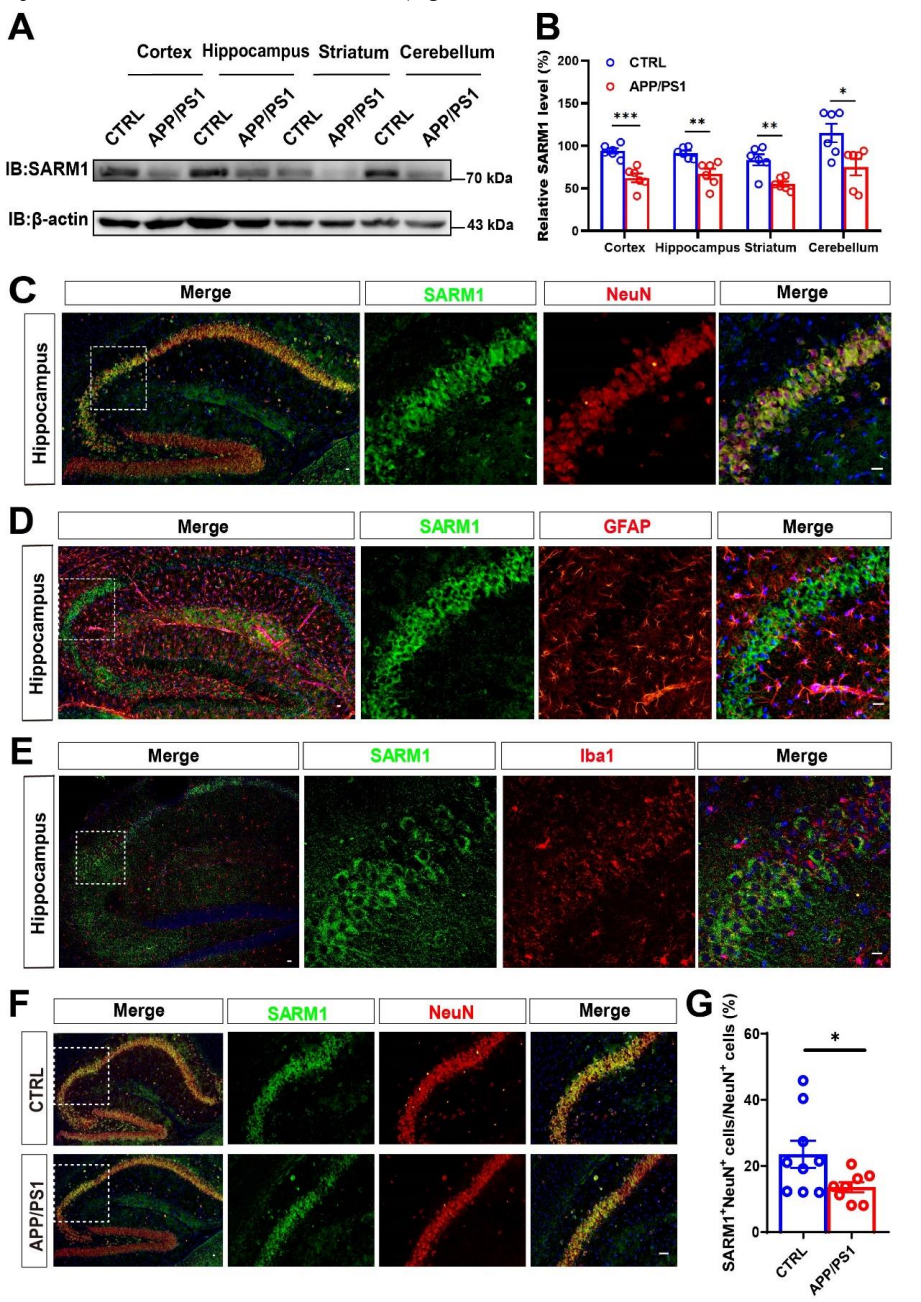
**SARM1 was mainly expressed in neurons and reduced in the hippocampal neurons of AD mice**. (**A**) Western blot analysis of SARM1 in the cortex, hippocampus, striatum and cerebellum of 9-month-old WT mice and APP/PS1 mice. (**B**) Quantification of the relative level of SARM1 in various brain regions of both WT and APP/PS1 groups, as shown in (A) (n = 6 blots from 3 mice per group). (C-E) Double immunostaining of SARM1 (green) and NeuN (red) (C), or SARM1 (green) and GFAP (red) (D), or SARM1 (green) and Iba1 (red) (E) in hippocampus of 9-month-old WT mice. (**F**) Double immunostaining for SARM1 (green) and NeuN (red) in the hippocampus of 9-month-old WT mice and APP/PS1 mice. (**G**) Quantitative analysis of the percentage of both SARM1^+^ and NeuN^+^ double positive over total NeuN^+^ cells were shown in (F) (n = 9 sections from 3 mice per group). Images of selected regions were shown at higher magnification. Data were mean ± SEM, ^*^P < 0.05, ^**^P < 0.01, ^***^P < 0.001, one-way ANOVA with Tukey’s post hoc analysis, compared with the control group. Scale bars, 20 μm.

To explore the potential role of SARM1 in AD, we examined the spatiotemporal expression pattern of SARM1 using the APP/PS1 dual transgenic mouse model, which is a common and typical model of AD [40]. We collected protein from various brain regions including the cortex, hippocampus, striatum, and cerebellum, and analyzed the SARM1 expression using western blot. As shown in Figure 1A, B, SARM1 was widely expressed in these brain regions and was significantly reduced in the AD model mice, compared with that in control mice. To further examine the cellular expression pattern of SARM1 in the hippocampus, double immunostaining was performed and showed that SARM1 was mainly expressed in NeuN^+^ neurons (Fig. 1C), lower expressed in GFAP^+^ astrocytes (Fig. 1D) and was weakly or not expressed in Iba1^+^ microglia (Fig. 1E). Moreover, the expression of SARM1 was significantly decreased in hippocampal neurons of APP/PS1 AD model mice (Fig. 1F, G). These results collectively suggested that SARM1 expression was reduced in hippocampal neurons of AD model mice.

### SARM1 deletion in CNS alleviated the memory deficits in AD model mice

To examine the potential role of SARM1 in AD, APP/PS1;SARM1^Nestin^-CKO mice were generated, in which the SARM1 gene was conditionally deleted in the CNS under AD background (Supplementary Fig. 1A). The AD phenotypes were confirmed in these mice by genotypic identification, as well as Barnes maze and Thioflavin S staining (Supplementary Fig. 1B-F). Again, SARM1 expression was significantly reduced in the cortex and hippocampus of SARM1^Nestin^-CKO mice and APP/PS1;SARM1^Nestin^-CKO mice, compared with that in control mice (Supplementary Fig. 1G-L). These results suggested the SARM1 was conditionally knockout in the CNS with the AD background (APP/PS1;SARM1^Nestin^-CKO mice).

**Figure 2.**
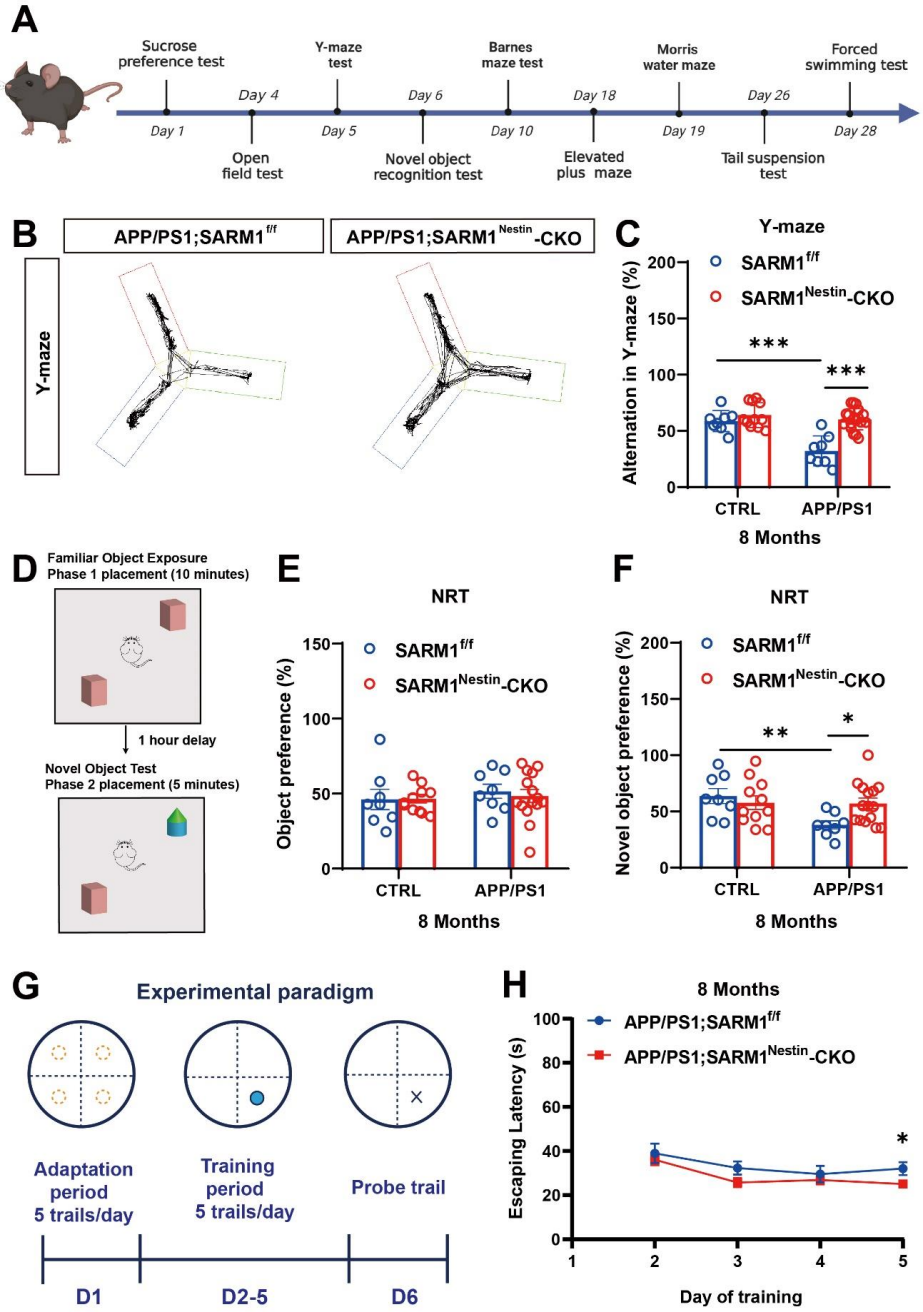
**SARM1 deletion in the CNS alleviated the memory deficits in AD model mice at 8 months of age**. (**A**) Schedule of evaluation of behavior tests. (**B**) Y-maze showing locomotor trajectories of 8-month-old APP/PS1;SARM1^f/f^ mice and APP/PS1;SARM1^Nestin^-CKO mice during the test phase. (**C**) Quantitative analysis of spontaneous alternation rate of 8-month-old SARM1^f/f^ mice (n = 8 mice), SARM1^Nestin^-CKO mice (n = 11 mice), APP/PS1;SARM1^f/f^ mice (n = 8 mice) and APP/PS1;SARM1^Nestin^-CKO mice (n = 16 mice) in the Y-maze. (**D**) Schematic diagram of the NRT in the test phase. (E-F) Quantitative analysis of the object preferences in 8-month-old SARM1^f/f^ mice (n = 8 mice), SARM1^Nestin^-CKO mice (n = 11 mice), APP/PS1;SARM1^f/f^ mice (n = 8 mice) and APP/PS1;SARM1^Nestin^-CKO mice (n = 15 mice) in the first phase (E) and the second phase (F) of the NRT. (**G**) Experimental paradigm of the MWM. (**H**) Quantitative analysis of escaping latency of 8-month-old APP/PS1;SARM1^f/f^ mice (n = 12 mice per group) and APP/PS1;SARM1^Nestin^-CKO (n = 14 mice) mice in the hidden platform tests. Data were mean ± SEM, ^*^P < 0.05, ^**^P < 0.01, ^***^P < 0.001, one-way ANOVA with Tukey’s post hoc analysis, compared with the control group.

We next examined whether SARM1 deletion affected the memory and cognitive function in AD. It is worth noting that cognitive decline in APP/PS1 mice usually starts at around 6 months old [41]. Interestingly, the memory deficits were alleviated in 8-month-old APP/PS1;SARM1^Nestin^-CKO mice, compared with that in 8-month-old APP/PS1;SARM1^f/f^ mice (Fig. 2B, C). In NRT assays, a preference for new objects was reduced in 8-month-old APP/PS1;SARM1^f/f^ mice, while 8-month-old APP/PS1;SARM1^Nestin^-CKO mice showed no significant change in preference for new objects (Fig. 2D-F). Furthermore, SARM1 deletion in CNS alleviated the spatial memory deficits in 8-month-old APP/PS1 model mice in the MWM test (Fig. 2G, H). These results suggested SARM1 deletion in CNS alleviated the memory deficits at late stage of AD model mice. However, no significant alteration between 3-month-old APP/PS1;SARM1^f/f^ mice and APP/PS1;SARM1^Nestin^-CKO mice was observed in memory-related phenotypes, including the latency in the MWM test and the percentage of spontaneous alternations in the Y-maze experiment (Supplementary Fig. 2A-D), suggesting SARM1 deletion did not affect the cognitive in AD model mice at an early stage. Taken together, these results of all behavioral tests above suggested that the memory impairment was alleviated in 8-month-old APP/PS1;SARM1^Nestin^-CKO mice, but no significant effects on 3-month-old mice.

### SARM1 deletion in CNS did not induce the anxiety or depression-like behavioral phenotypes

Anxiety and depression are commonly observed in patients with AD [42]. To examine the role of SARM1 in CNS with regard to anxiety and depression-like phenotypes, the OFT and EPM tests were performed. The results showed no significant differences in the time spent in the central region of the OFT and the distance of movement between 3-month-old APP/PS1;SARM1^f/f^ mice and 3-month-old APP/PS1;SARM1^Nestin^-CKO mice (Supplementary Fig. 3A-C), and between 8-month-old APP/PS1;SARM1^f/f^ mice and 8-month-old APP/PS1; SARM1^Nestin^-CKO mice (Supplementary Fig. 4A-D). These results suggested that SARM1 deletion in CNS did not induce anxiety or depression-like behavioral phenotypes. These findings were further confirmed by the results of the EPM test (Supplementary Fig. 3D-G, Supplementary Fig. 4E, F).

Subsequently, we examined the depressive-like behaviors in these mice by the SPT, FST and TST. Interestingly, there was no significant difference in the preference index for sucrose among the four groups of 3-month-old mice in the SPT (Supplementary Fig. 5A-D). However, 8-months-old the APP/PS1;SARM1^f/f^ mice exhibited a significant reduction in the preference index for sucrose, indicating pronounced depression-like behaviors. In contrast, there was no significant difference in depression-like behaviors between the APP/ PS1;SARM1^Nestin^-CKO mice and the APP/ PS1;SARM1^f/f^ mice (Supplementary Fig. 6A). To further confirm this observation, both TST and FST tests also showed no significant difference in the percentage of immobility time among the four groups of mice at 3-month-old (Supplementary Fig. 5E, F) and 8-month-old (Supplementary Fig. 6B-D), suggesting that the depression-like behaviors was not observed after deletion of SARM1 in CNS. Taken together, these results suggested that SARM1 deletion in the CNS did not result in the anxiety or depression-like behavioral phenotypes.

### SARM1 deletion in CNS reduced the Aβ deposition in AD model mice

**Figure 3.**
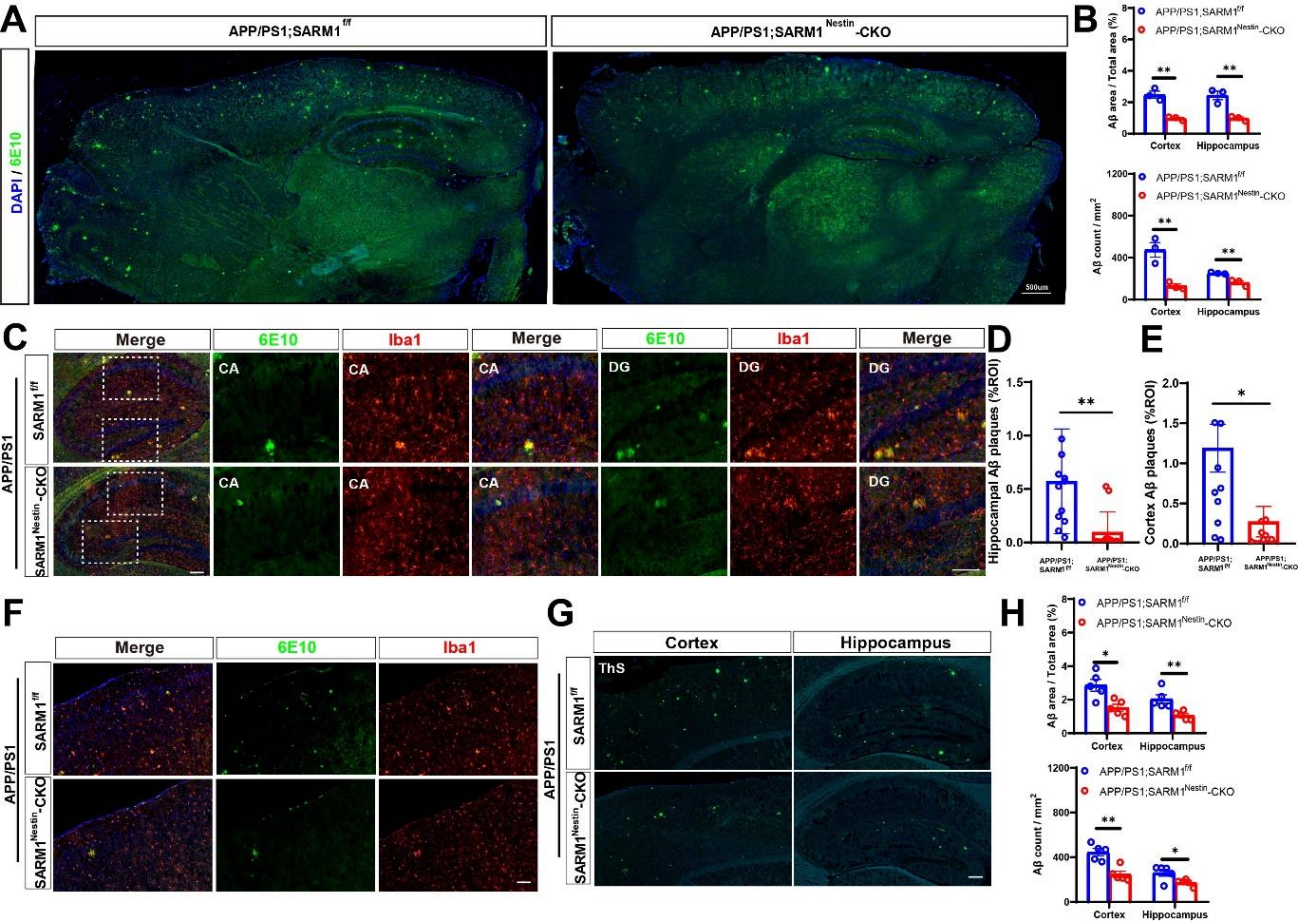
**SARM1 deletion in CNS reduced Aβ deposition of AD model mice**. (**A**) Immunostaining of 6E10 (green) in whole brain regions of 9-month-old APP/PS1;SARM1^f/f^ mice and APP/PS1;SARM1^Nestin^-CKO mice. (**B**) Quantitative analysis of the percentage and the count of Aβ plaques in the cortex and hippocampus, as shown in (A) (n = 3 sections from 3 mice per group). (**C**) Double immunostaining of 6E10 (green) and Iba1 (red) in the hippocampus of 9-month-old APP/PS1;SARM1^f/f^ mice and APP/PS1;SARM1^Nestin^-CKO mice. (**D**) Quantitative analysis of the percentage of Aβ plaque area in the hippocampus, as shown in (C) (n = 12 sections from 4 mice per group). (**E**) Quantitative analysis of the percentage of Aβ plaque area in the cortex, as shown in (F) (n = 12 sections from 4 mice per group). (**F**) Double immunostaining of 6E10 (green) and Iba1 (red) in the cortex of 9-month-old APP/PS1;SARM1^f/f^ mice and APP/PS1;SARM1^Nestin^-CKO mice. (**G**) Thioflavin S staining showed Aβ deposition in the cortex and hippocampus of 9-month-old APP/PS1;SARM1^f/f^ mice and APP/PS1;SARM1^Nestin^-CKO mice. (**H**) Quantitative analysis of the percentage and the count of Aβ plaques in the cortex and hippocampus, as shown in (G) (n = 5 sections from 5 mice per group). Images of selected regions were shown at higher magnification. Data were mean ± SEM, ^*^P < 0.05, ^**^P < 0.01, one-way ANOVA with Bonferroni (B) / Tukey’s (D, E, H) post hoc analysis, compared with the control group. Scale bars, 20 μm.

Next, we performed immunostaining using the 6E10 biomarker to detect Aβ deposition in AD model mice. As shown in Figure 3A-F, a significant reduction in Aβ deposition in the 9-month-old APP/PS1;SARM1^Nestin^-CKO mice, with microglia aggregating around the plaques (Fig. 3C-F), compared with that in 9-month-old APP/PS1;SARM1^f/f^ mice. Moreover, Thioflavin S staining further showed Aβ deposition was decreased both in the hippocampus and cortex of 9-month-old APP/PS1;SARM1^Nestin^-CKO mice (Fig. 3G-H). Taken together, these results suggested that SARM1 deletion in CNS reduced the Aβ deposition in AD model mice.

**Figure 4.**
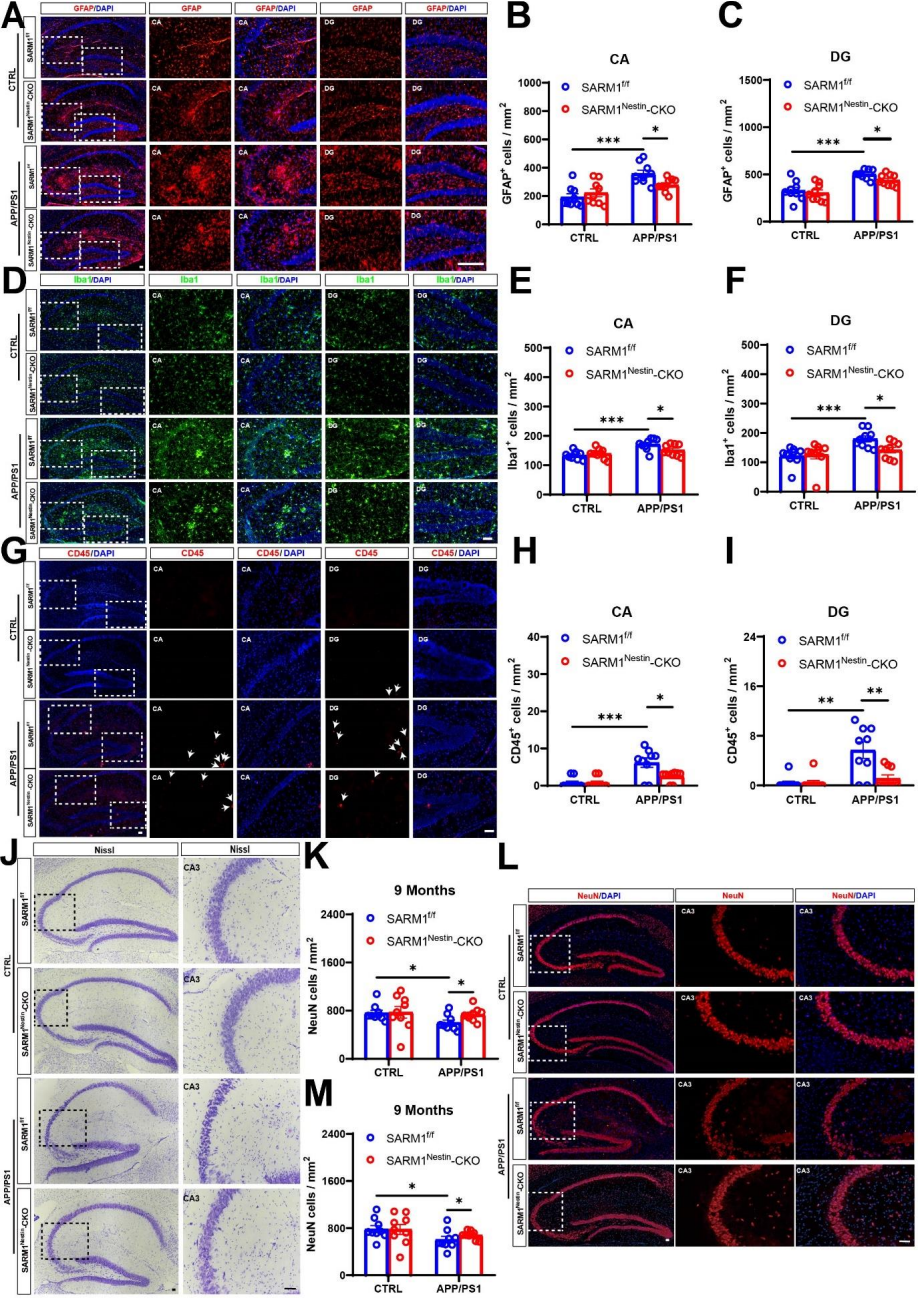
**Inflammatory infiltration and neuronal loss were reduced in APP/PS1;SARM1^Nestin^-CKO mice**. (**A**) Immunostaining of GFAP (red) in the hippocampus of 9-month-old SARM1^f/f^ mice, SARM1^Nestin^-CKO mice, APP/PS1;SARM1^f/f^ mice and APP/PS1;SARM1^Nestin^-CKO mice. (B-C) Quantitative analysis of the density of GFAP^+^ cells in the CA (B) and DG (C) regions as shown in (A) (n = 9 sections from 9 mice per group). (**D**) Immunostaining of Iba1 (green) in the hippocampus of 9-month-old SARM1^f/f^ mice, SARM1^Nestin^-CKO mice, APP/PS1;SARM1^f/f^ mice and APP/PS1;SARM1^Nestin^-CKO mice. (E-F) Quantitative analysis of the density of Iba1^+^ cells in the CA (E) and DG (F) regions as shown in (D) (n = 9 sections from 9 mice per group). (**G**) Immunostaining of CD45 (red) in the hippocampus of 9-month-old SARM1^f/f^ mice, SARM1^Nestin^-CKO mice, APP/PS1;SARM1^f/f^ mice and APP/PS1;SARM1^Nestin^-CKO mice. (H-I) Quantitative analysis of the density of CD45^+^ cells in the CA (H) and DG (I) regions as shown in (G) (n = 9 sections from 9 mice per group). (**J**) Nissl staining in the hippocampus of 9-month-old SARM1^f/f^ mice, SARM1^Nestin^-CKO mice, APP/PS1;SARM1^f/f^ mice and APP/PS1;SARM1^Nestin^-CKO mice. (**K**) Quantitative analysis of the density of NeuN^+^ cells in the hippocampus as shown in (J) (n = 9 sections from 9 mice per group). (**L**) Immunostaining of NeuN (red) in the hippocampus of 9-month-old SARM1^f/f^ mice, SARM1^Nestin^-CKO mice, APP/PS1;SARM1^f/f^ mice and APP/PS1;SARM1^Nestin^-CKO mice. (**M**) Quantitative analysis of the density of NeuN^+^ cells in the hippocampus as shown in (L) (n = 9 sections from 9 mice per group). Images of selected regions were shown at higher magnification. Data were mean ± SEM, ^*^P < 0.05, ^**^P < 0.01, ^***^P < 0.001, one-way ANOVA with Tukey’s post hoc analysis, compared with the control group. Scale bars, 20 μm.

### SARM1 deletion in CNS inhibited the neuro-inflammatory response and loss of neurons in the AD model mice

Previous studies have shown that neuroinflammatory responses are present in AD mice [43]. Thus, we examined whether neuroinflammation was affected after SARM1 deletion in CNS with AD background. As shown in Figure 4A-C, the density of GFAP^+^ astrocytes in the CA and DG region was reduced in the hippocampus of 9-month-old APP/PS1;SARM1^Nestin^-CKO mice, compared with that in APP/PS1;SARM1^f/f^ mice. Additionally, the densities of Iba1^+^ microglia and CD45^+^ (a marker of T cells) cells were significantly decreased in both the DG and CA regions of 9-month-old APP/PS1;SARM1^Nestin^-CKO mice (Fig. 4D-I). However, there was no significant difference in the density and morphology of hippocampal inflammatory cells between SARM1^Nestin^-CKO mice and SARM1^f/f^ mice (Fig. 4A-I), suggesting that SARM1 deletion in CNS might not affect the development of astrocytes and microglia. In summary, these results suggested that SARM1 deletion in CNS inhibited inflammatory infiltration in AD model mice.

We next examined the neuronal loss at different ages in APP/PS1;SARM1^Nestin^-CKO mice. Nissl staining and immunostaining showed that neuronal loss was attenuated in the hippocampus of the 9-month-old APP/PS1; SARM1^Nestin^-CKO mice, compared with that in APP/PS1;SARM1^f/f^ mice (Fig. 4J-M). Taken together, these results suggested that SARM1 deletion in CNS inhibited inflammatory infiltration and neuronal loss in the middle and late stages of APP/PS1 AD model mice.

### SARM1 deletion in CNS alleviated the cognitive decline, Aβ deposition and inflammatory infiltration in AD model mice by downregulating TNF-α signaling

**Figure 5.**
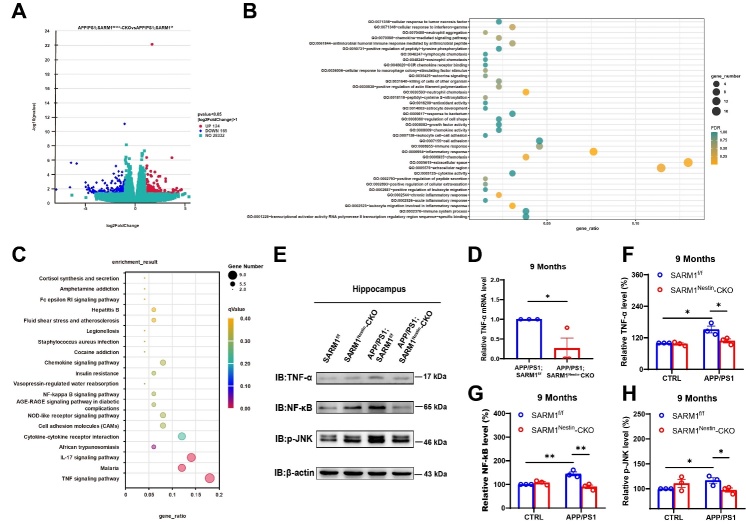
**RNA-seq-based enrichment analysis of hippocampal tissues from 9-month-old APP/PS1;SARM1^f/f^ mice and APP/PS1;SARM1^Nestin^-CKO mice**. (**A**) RNA-seq-based enrichment analysis of the hippocampus of 9-month-old APP/PS1;SARM1^f/f^ mice and APP/PS1;SARM1^Nestin^-CKO mice. (**B**) Enrichment map for GO analysis. (**C**) KEGG enrichment analysis showed the highest score of the TNF-α signaling pathway. (**D**) The expression of TNF-α in the hippocampus of 9-month-old APP/PS1;SARM1^f/f^ mice and APP/PS1;SARM1^Nestin^-CKO mice was detected by qPCR (n = 3 mice per group, normalized to APP/PS1;SARM1^f/f^ mice). (**E**) Western blot detected the expression levels of TNF-α, NF-κB and p-JNK in the hippocampus of 9-month-old SARM1^f/f^ mice, SARM1^Nestin^-CKO mice, APP/PS1;SARM1^f/f^ mice and APP/PS1;SARM1^Nestin^-CKO mice. (F-H) Quantification of the relative expression levels of TNF-α (F), NF-κB (G) and p-JNK (H) as shown in (E) (n = 3 mice per group, normalized to SARM1^f/f^ mice). Data were mean ± SEM, ^*^P < 0.05, ^**^P < 0.01, one-way ANOVA with Bonferroni post hoc analysis, compared with the control group.

How did SARM1 deletion lead to a reduction in neuroinflammation in AD model mice? To address this question, mRNA sequencing was performed in hippocampal tissues from both APP/PS1;SARM1^f/f^ mice and APP/PS1;SARM1^Nestin^-CKO mice. As shown in Figure 5A, we found that 124 genes were upregulated and 165 genes were down-regulated in APP/PS1;SARM1^Nestin^-CKO mice. To examine the functional relevance of these genes, GO and KEGG enrichment analyses were performed in all expressed genes using the hallmark gene set, and revealed that a functional association between SARM1 and TNF-α signaling (Fig. 5B, C). Interestingly, previous studies have reported that TNF-α signaling is strongly associated with inflammation and plays a crucial role in AD [44]. The downregulation of TNF-α pathway was confirmed by qRT-PCR and western blot assays in the hippocampus tissues of 9-month-old APP/PS1;SARM1^Nestin^-CKO mice (Fig. 5D-H). As expected, there was no significant difference in the expression levels of the TNF-α related factors between SARM1^f/f^ and SARM1^Nestin^-CKO mice (Fig. 5E-H). These results suggested that SARM1 deletion in CNS resulted into the downregulation of the TNF-α signaling pathway, which contributed to the reduction of neuroinflammatory infiltration, Aβ deposition, and neuronal loss in APP/PS1 model mice.

**Figure 6.**
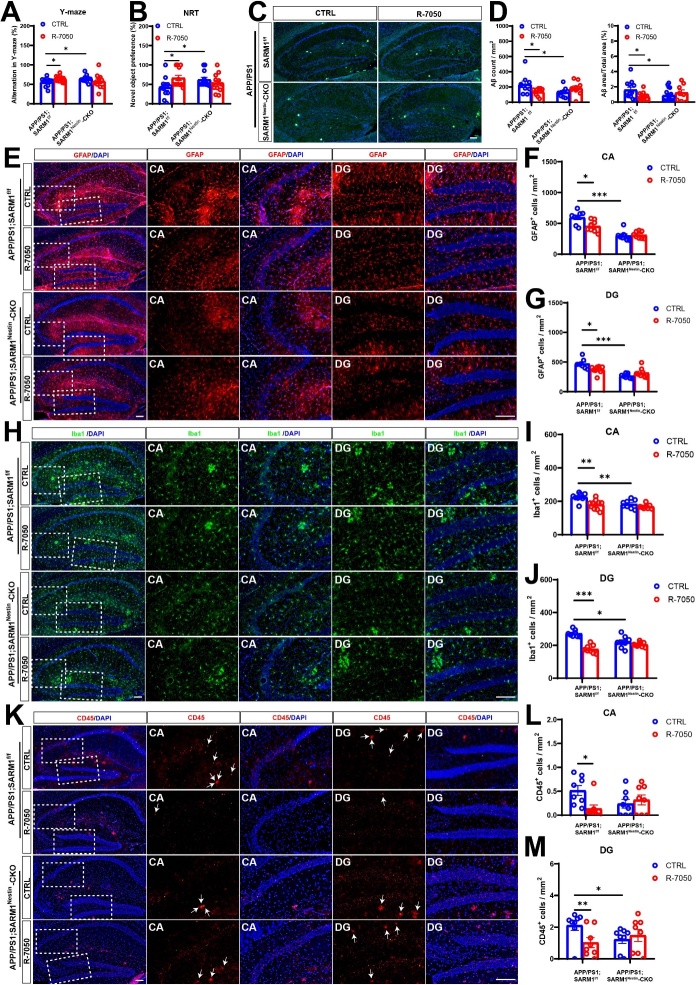
**SARM1 promoted memory impairment, Aβ deposition and inflammatory infiltration in AD model mice through TNF-α signaling**. (**A**) Quantification of spontaneous alternation rates in the Y-maze at 8-month-old vehicle-treated APP/PS1;SARM1^f/f^ mice, R-7050-treated APP/PS1;SARM1^f/f^ mice, vehicle-treated APP/PS1;SARM1^Nestin^-CKO mice and R-7050-treated APP/PS1;SARM1^Nestin^-CKO mice (n = 12 mice per group). (**B**) Quantification of the object preferences in 8-month-old vehicle-treated APP/PS1;SARM1^f/f^ mice, R-7050-treated APP/PS1;SARM1^f/f^ mice, vehicle-treated APP/PS1;SARM1^Nestin^-CKO mice and R-7050-treated APP/PS1;SARM1^Nestin^-CKO mice (n = 12 mice per group) during the second phase of the NRT. (**C**) Thioflavin S staining showed Aβ deposition in the hippocampus of 9-month-old vehicle, R-7050-treated SARM1^f/f^ and SARM1^Nestin^-CKO APP/PS1 mice. (**D**) Quantitative analysis of the count and the percentage of Aβ plaques in the hippocampus, as shown in (C) (n = 9 sections from 3 mice per group). (**E**) Immunostaining of GFAP (red) in the hippocampus of 9-month-old vehicle, R-7050-treated SARM1^f/f^ and SARM1^Nestin^-CKO APP/PS1 mice. (F-G) Quantitative analysis of the density of GFAP^+^ cells in the CA (F) and DG (G) regions as shown in (E) (n = 8 sections from 8 mice per group). (**H**) Immunostaining of hippocampal Iba1 (green) in vehicle, R-7050-treated SARM1^f/f^ and SARM1^Nestin^-CKO APP/PS1 mice. (I-J) Quantitative analysis of the density of Iba1^+^ cells in the CA (I) and DG (J) regions as shown in (H) (n = 8 sections from 8 mice per group). (**K**) Immunostaining for CD45 (red) in the hippocampus of 9-month-old vehicle, R-7050-treated SARM1^f/f^ and SARM1^Nestin^-CKO APP/PS1 mice. (L-M) Quantitative analysis of the density of CD45^+^ cells in the CA (L) and DG (M) regions as shown in (K) (n = 8 sections from 8 mice per group). The image of the selected area is displayed at a higher magnification. Data are mean ± SEM, ^*^P < 0.05, ^**^P < 0.01, ^***^P < 0.001, one-way ANOVA with Tukey’s post hoc analysis, compared with the control group. Scale bar, 20 μm.

To further confirm the role of the SARM1-TNF-α pathway in AD, two groups of mice, APP/PS1;SARM1^f/f^ and APP/PS1;SARM1^Nestin^-CKO mice were treated with the TNF-α inhibitor R-7050 through intraperitoneal injection. The memory-related phenotypes were observed by Y-maze test, R-7050-treated APP/PS1;SARM1^f/f^ mice had an increased percentage of spontaneous alternations, compared with that in control group, while there was no significant difference in the percentage of spontaneous alternations between vehicle-treated and R-7050-treated APP/PS1;SARM1^Nestin^-CKO mice (Fig. 6A). Similarly, during the second phase of NRT, APP/PS1;SARM1^f/f^ mice after R-7050 treatment showed an increased preference for novel objects, compared with that in vehicle-treated APP/PS1;SARM1^f/f^ mice. However, CTRL;APP/PS1;SARM1^Nestin^-CKO and R-7050;APP/ PS1;SARM1^Nestin^-CKO mice did not show significant change in their preference for novel objects (Fig. 6B). These results indicated that TNF-α inhibitor improved the memory impairment of AD mice to a certain extent. Furthermore, as shown in Figure 6C, D, Thioflavin S staining showed Aβ deposition was reduced in the hippocampus of R-7050;APP/PS1;SARM1^f/f^ mice, compared with that CTRL;APP/PS1;SARM1^f/f^ mice. There was no significant difference in Aβ deposition in the hippocampus of R-7050;APP/PS1;SARM1^Nestin^-CKO and CTRL;APP/PS1;SARM1^Nestin^-CKO mice (Fig. 6C, D).

Next, we examined whether hippocampal neuroinflammation was altered in different groups. R-7050 treatment significantly reduced the density of GFAP^+^ astrocytes in the hippocampal CA and DG regions in APP/PS1 model mice, compared with that in vehicle-treated SARM1^f/f^ mice, exactly as expected. However, there was no significant difference in the DG and CA regions of the vehicle-treated and R-7050-treated APP/PS1;SARM1^Nestin^-CKO mice (Fig. 6E-G). Consistent with the results of GFAP^+^ astrocytes, the density of Iba1^+^ cells (Fig. 6H-J) and CD45^+^ cells (Fig. 6K-M) also were reduced in whole hippocampus of R-7050-treated APP/PS1;SARM1^f/f^ mice, compared with APP/PS1;SARM1^f/f^ mice treated with normal saline. Whereas the density of Iba1 (Fig. 6H-J) and CD45 (Fig. 6K-M) in the DG and CA regions had no obvious difference between vehicle-treated and R-7050-treated 9-month-old APP/PS1;SARM1^Nestin^-CKO mice. Taken together, these results suggested that SARM1 deletion in CNS alleviated cognitive decline, Aβ deposition and inflammatory infiltration in APP/PS1 mice by downregulation of TNF-α signaling.

## DISCUSSION

In this study, we examined the role and mechanism of SARM1 in the pathogenesis of AD. Our findings revealed that neuronal SARM1 expression was reduced in the APP/PS1 model mice, and SARM1 deletion in CNS delayed the cognitive decline in APP/PS1 AD model mice. Additionally, SARM1 deletion in CNS reduced inflammatory infiltration and amyloid deposition in the APP/PS1 AD model mice by inhibiting TNF-α signaling (see Model Diagram: Graphical Abstract Image).

Consistent with previous study [45], the present study found that SARM1 was mainly expressed in brain neurons, and the expression of neuronal SARM1 was decreased in 9-month-old APP/PS1 model mice (Fig. 1). Our previous studies have shown that SARM1 expression is increased in acute spinal cord injury [46]. This may suggest that SARM1 plays different roles in different disease models. Some previous studies have shown that in SARM1 knockdown mice, dendritic arbors of neurons were less complex, and suggested that SARM1 is required for proper initiation and elongation of dendrites, axonal outgrowth, and neuronal polarization [47]. Our previous study has shown that parvalbumin-positive interneurons (PVI)-specific conditional SARM1 deletion (SARM1^PV^-CKO) mice exhibited autism-like behaviors and a reduction in the number of PVIs due to apoptosis, suggesting that SARM1 deficiency in PVIs may contribute to the pathogenesis of autism spectrum disorder [45]. However, our recent studies suggested that there was no significant difference in the neuronal number and distribution in the spinal cords between SARM1^f/f^ and SARM1^Nestin^-CKO mice. Moreover, we also found that SARM1 deletion in CNS did not affect the number of neurons in the brain (Fig. 4). Furthermore, there were no significant different in several behavioral tests such as OFT, SPT, FST, between SARM1^f/f^ and SARM1^Nestin^-CKO mice, suggesting that SARM1 deletion in CNS may not affect the development of spinal cords and brains, and normal functions. However, whether SARM1 affects the integrity or plasticity of neurons in more detail needs further studies in the future.

But, intriguingly, our studies showed the deletion of SARM1 in the AD model mice could alleviate Aβ deposition to some extent, thereby preventing cognitive impairment (Fig. 2 and Fig. 3), which was consistent with previous studies that have demonstrated the negative impact of SARM1 on cognitive impairment in various diseases such as traumatic brain injury and postoperative cognitive dysfunction [18]. Furthermore, we observed that the deletion of SARM1 in 8-month-old, rather than 3-month-old APP/PS1 mice ameliorated disease progression of AD as evidenced by behavioral and histological tests (Fig. 2, 3, 4, and Supplementary Fig. 2). AD is characterized by progressive, age-dependent degeneration of neurons in CNS, and axonal degeneration is a prominent feature of late AD models [48]. A large body of evidence indicates that SARM1 is the central executioner of the axonal degeneration pathway that culminates in axonal depletion [49, 50]. These may be the main reason why SARM1 has a greater impact on APP/PS1 mice in the late-life stages. Apart from the above, 9-month-old APP/PS1 model mice showed decrease in the expression of SARM1 (Fig. 1), which may further indicate the effect of SARM1 in APP/PS1 mice in the late stage.

However, these results from the SARM1-knockout mice and the SARM1 expression in APP/PS1 mice seem contradictive. We speculated that the expression of SARM1 is decreased in AD model mice, which may be a self-protection or compensatory mechanism of AD model mice. Nevertheless, compared with the control group, the expression level of SARM1 in 9-month-old APP/PS1 mice decreased mildly in the hippocampus, which may lead to limited self-protective capacity. However, as shown in Supplementary Fig. 1, the western blot showing conditional SARM1 knockout efficiency revealed a mean knockout efficiency of 72 ± 4%, which may produce superimposed effects. Additionally, it has been shown that overexpression of SARM1 induced some cell death and significantly increased cytotoxicity [51], which is the consistent with results from SARM1-knockout mice. To confirm the effect of SARM1 in AD, an overexpression vector of SARM1 will be constructed to further explore the function of SARM1 for AD both *in vitro* and *in vivo*.

Moreover, SARM1 deletion in CNS reduced the infiltration of inflammatory cells such as glial cells in the CA3 region of the hippocampus, which was accompanied by the loss of neurons (Fig. 4). Previous studies have shown that neuronal SARM1 may produce the inflammatory cytokines and chemokines, which may play key roles in glial cell-mediated neuroinflammatory response [52, 53]. Whereas, interestingly, some studies have suggested that SARM1 deficiency may lead to impairment in glial activation [54], for example, after mild traumatic brain injury, SARM1 deletion reduced astrocytic and microglial activation to induce significantly better motor and cognitive performance [18]. However, as shown in Figure 5, we also found that some important inflammatory cytokines, including TNF-α and NF-κB, were decreased after the deletion of SARM1 in AD model mice, which indicated that the impairment of glial activation in neuronal SARM1-deficient AD model mice is likely to be the response to inflammatory factors. Rather, it is more likely that the neuronal immune response influences the activation of glia. However, these two possibilities are not mutually exclusive, and more investigation is needed to address the detailed regulation in the future.

How does SARM1 in neurons affect AD disease progression? TNF-α is an important mediator of the inflammatory response, expressed in neurons and glial cells in the CNS, which promotes inflammatory response by recruiting glial cells to the lesion site and leads to glial activation [55]. Studies have shown that the TNF-α signaling pathway plays an important role in immune inflammation of neurodegenerative pathologies. For example, the elevated TNF-α levels at the lesion site were observed in multiple sclerosis and Parkinson’s disease patients at autopsy, and TNF-α was also significantly up-regulated in AD patients and AD model mice [56, 57]. Consistent with these previous studies, we found that the TNF-α signaling pathway was upregulated in APP/PS1 model mice (Fig. 5). Interestingly, our RNA-seq results showed that the TNF-α signaling pathway was significantly reduced in APP/PS1;SARM1^Nestin^-CKO mice, compared to that in APP/PS1;SARM1^f/f^ mice (Fig. 5). We used the TNF-α inhibitor, R-7050, to further verify the relationship between SARM1 and TNF-α in AD. We found that the use of R-7050 on APP/PS1 mice further reduced glial cells infiltration and improved the memory function of APP/PS1 model mice as expected. This is consistent with previous studies that showed inhibition of TNF-α signaling in AD model mice prevents working memory deficits as measured by the Y-maze test, and improved long-term memory deficits [54]. Additionally, the use of TNF-α inhibitor could lead to reduction of Aβ deposition in AD model mice and alleviate brain inflammation [55]. However, after knockout SARM1 in CNS, there was no significant difference between vehicle-treated and R-7050-treated APP/PS1 mice, which could be key evidence that TNF-α may be downstream of SARM1 to affect the inflammatory infiltration and cognitive impairment in APP/PS1 model mice. In conclusion, our sequencing data and experimental results suggested that the function of SARM1 in AD model mice is partially dependent on TNF-related signaling pathways. Previous studies have shown that SARM1 in neurons attenuates the activity of TRIF in the TLR3 and TLR4 pathways in immune response, and expression of SARM1 reduces TRIF-induced NF-κB activation and cytokine production [58]. Meanwhile, in our previous study has shown that NF-κB may act downstream of SARM1 to regulate neuroinflammation at the early phase of SCI [21]. Therefore, we speculated that neuronal SARM1 may produce the inflammatory cytokines and chemokines, which may play key roles in glial cell-mediated neuroinflammatory response [52, 53].

In summary, our results suggest that SARM1 deletion could affect neuroinflammation by down-regulating the TNF-α signaling pathway, which in turn improves memory function in AD model mice. These results imply that SARM1 might promote inflammatory infiltration and plaque deposition in AD model mice, thereby negatively impacting AD-related neuro-degeneration. Our study not only highlights the previously unknown function of SARM1 signaling in AD but also establishes a link between SARM1 and TNF-α signaling, which could potentially serve as a novel therapeutic target for AD treatment by selectively inhibiting the function of SARM1-TNF-α signaling pathway.

## Supplementary Materials

The Supplementary data can be found online at: www.aginganddisease.org/EN/10.14336/AD.2023.0516-1.


