# Supplementary material for: SARM1 Promotes Neurodegeneration and Memory Impairment in Mouse Models of Alzheimer's Disease
Source: Aging Dis. 2024 Feb 1;15(1):390–407. doi: 10.14336/AD.2023.0516-1 (PMC10796105; doi:10.14336/AD.2023.0516-1)
Supplement: Supplementary file 1 [file AD-15-1-390-s.pdf]

SUPPLEMENTARY DATA

SARM1 Promotes Neurodegeneration and Memory Impairment in Mouse Models of Alzheimer's Disease

Xuemeng Miao, Qian Wu, Siyu Du, Ludan Xiang, Siyao Zhou, Junzhe Zhu, Zirun Chen, Hui Wang, Xuyi Pan, Yiren Fan, Lihan Zhang, Jingkang Qian, Yuxuan Xing, Yiyang Xie, Lixin Hu, Haiyun Xu, Wei Wang, Ying Wang, Zhihui Huang

© 2023. Miao X et al. Published online at http://www.aginganddisease.org/EN/10.14336/AD.2023.04516-1

SUPPLEMENTARY DATA

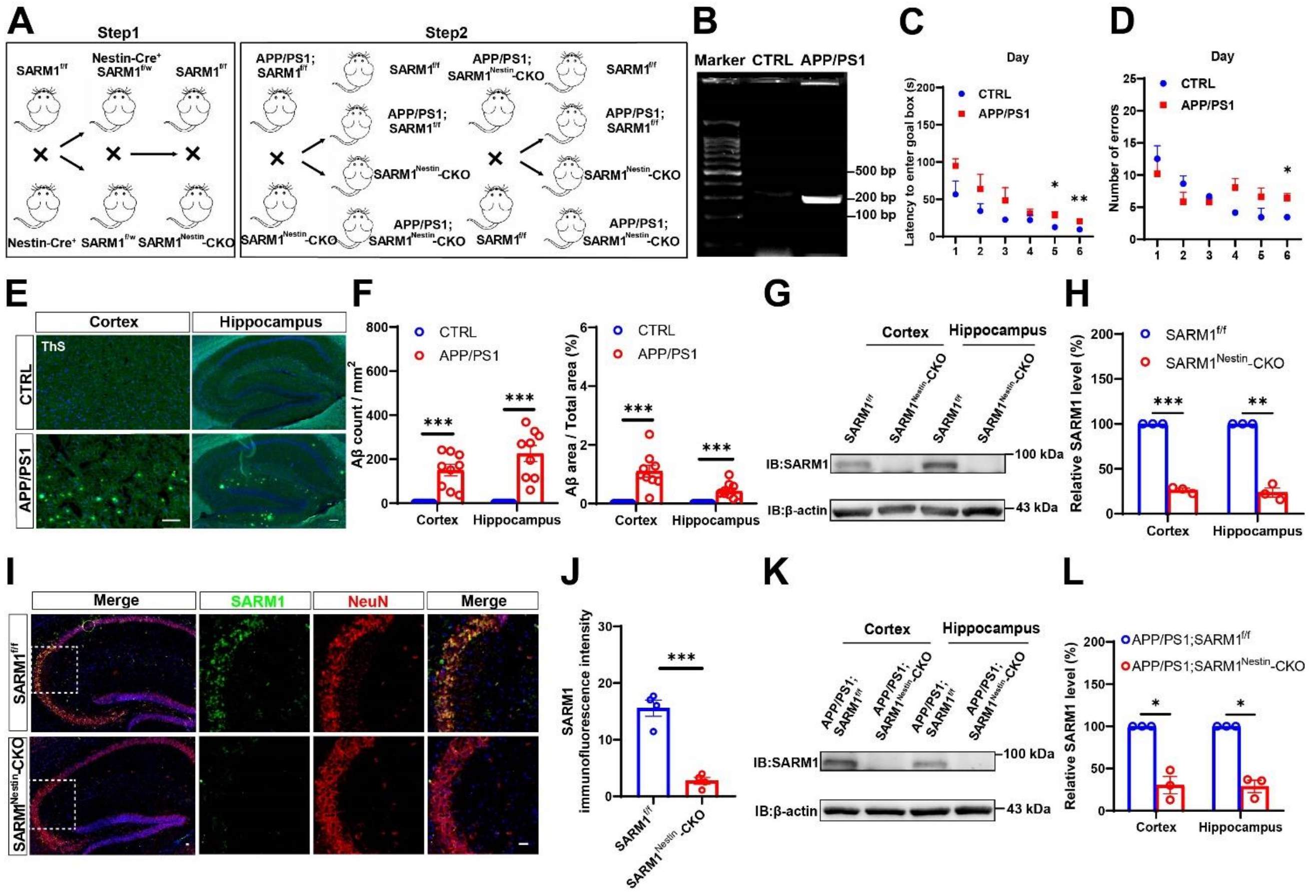

Supplementary Figure 1. Identification of APP/PS1;SARM1^{Nestin}-CKO mice. **(A)** Flow chart of the process of breeding APP/PS1;SARM1^{Nestin}-CKO mice and their littermate control. **(B)** Genotyping of APP/PS1 mice by agarose gel electrophoresis. **(C-D)** The memory analysis of 12-month-old SARM1$^{f/f}$ and APP/PS1;SARM1$^{f/f}$ mice in Barnes maze, and the latency to enter goal box assays (n = 12 mice per group). **(E)** Thioflavin S staining showed Aβ deposition in the cortex and hippocampus of 9-month-old SARM1$^{f/f}$ mice and APP/PS1;SARM1$^{f/f}$ mice. **(F)** Quantitative analysis of the count and the percentage of Aβ plaques in the cortex and hippocampus, as shown in (E) (n = 9 sections from 3 mice per group). **(G)** Western blot analysis of SARM1 in the hippocampus and cortex of 9-month-old SARM1$^{f/f}$ mice and SARM1^{Nestin}-CKO mice. **(H)** Quantification of the relative level of SARM1 as shown in (G) (n = 3 per group from 3 mice, normalized to SARM1$^{f/f}$ mice). **(I)** Double immunostaining of SARM1 (green) and NeuN (red) in the hippocampus of 9-month-old SARM1$^{f/f}$ mice and SARM1^{Nestin}-CKO mice. **(J)** Quantitative analysis of the relative SARM1 immunofluorescence intensity as shown in (I) (n = 4 per group from 4 mice). **(K)** Western blot analysis of SARM1 in the hippocampus and cortex of 9-month-old APP/PS1;SARM1$^{f/f}$ mice and APP/PS1;SARM1^{Nestin}-CKO mice. **(L)** Quantification of the relative level of SARM1 as shown in (K) (n = 3 per group from 3 mice, normalized to APP/PS1;SARM1$^{f/f}$ mice). Images of selected regions were shown at higher magnification. Data were mean ± SEM, $^{*}P < 0.05$, $^{**}P < 0.01$, $^{***}P < 0.001$, one-way ANOVA with Bonferroni (H, J, L) / Tukey's (C, D, F) post hoc analysis, compared with the control group. Scale bars, 20 μm.

© 2023. Miao X et al. Published online at http://www.aginganddisease.org/EN/10.14336/AD.2023.04516-1

SUPPLEMENTARY DATA

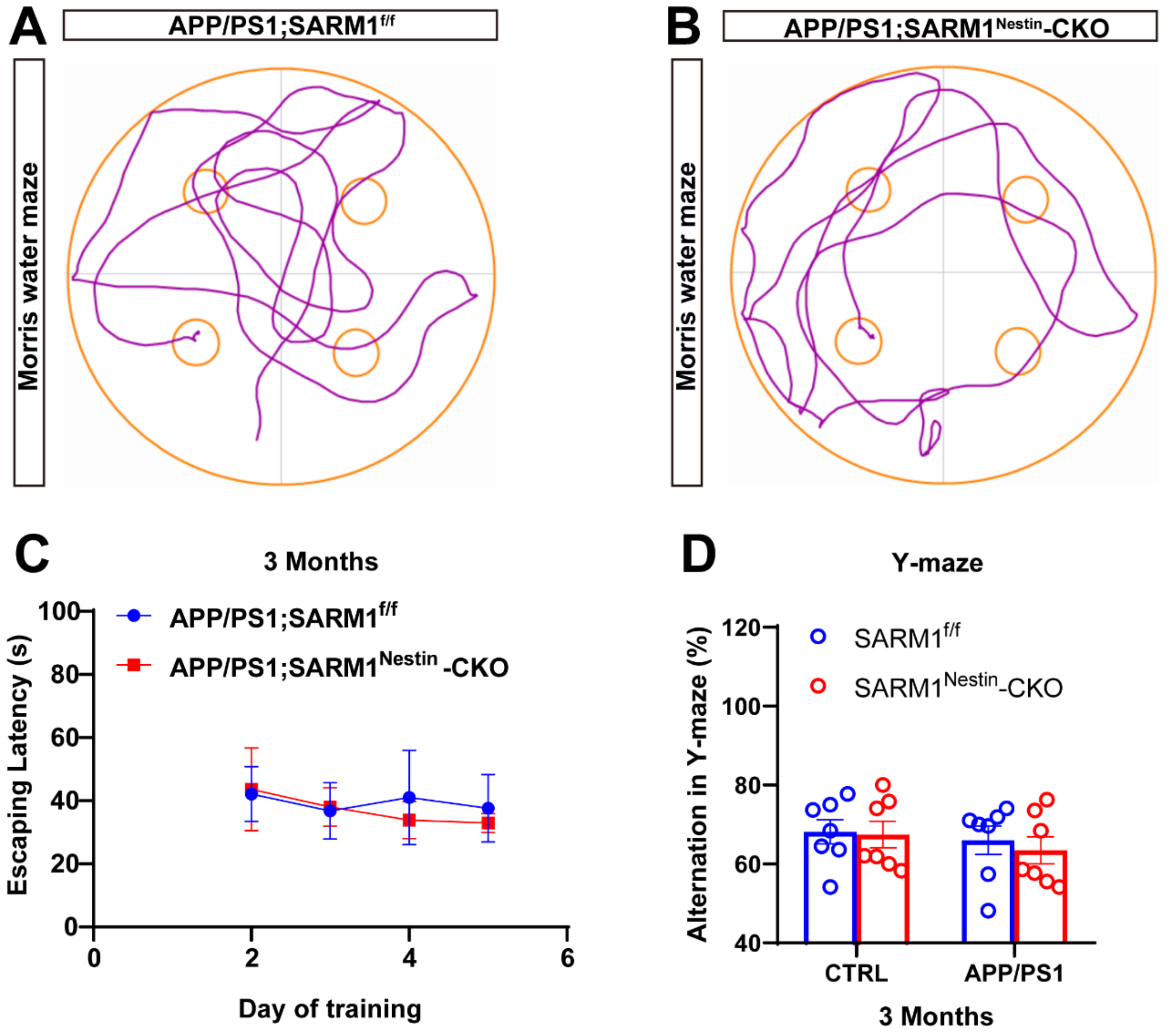

Supplementary Figure 2. APP/PS1;SARM1^{Nestin}-CKO mice had no memory impairment at 3-month-old. (A-B) Typical motor trajectories in MWM test in 3-month-old APP/PS1;SARM1$^{f/f}$ mice (A) and APP/PS1;SARM1^{Nestin}-CKO mice (B) (n = 7 mice per group). **(C)** Quantification of latency in the MWM test from day 2 to day 5 in 3-month-old APP/PS1;SARM1$^{f/f}$ mice, APP/PS1;SARM1^{Nestin}-CKO mice (n = 7 mice per group). **(D)** Quantification of the percentage of spontaneous alternation rate in the Y-maze test for 3-month-old SARM1$^{f/f}$ mice, SARM1^{Nestin}-CKO mice, APP/PS1;SARM1$^{f/f}$ mice and APP/PS1;SARM1^{Nestin}-CKO mice (n = 7 mice per group). Data were mean ± SEM, one-way ANOVA with Tukey's post hoc analysis, compared with the control group.

© 2023. Miao X et al. Published online at http://www.aginganddisease.org/EN/10.14336/AD.2023.04516-1

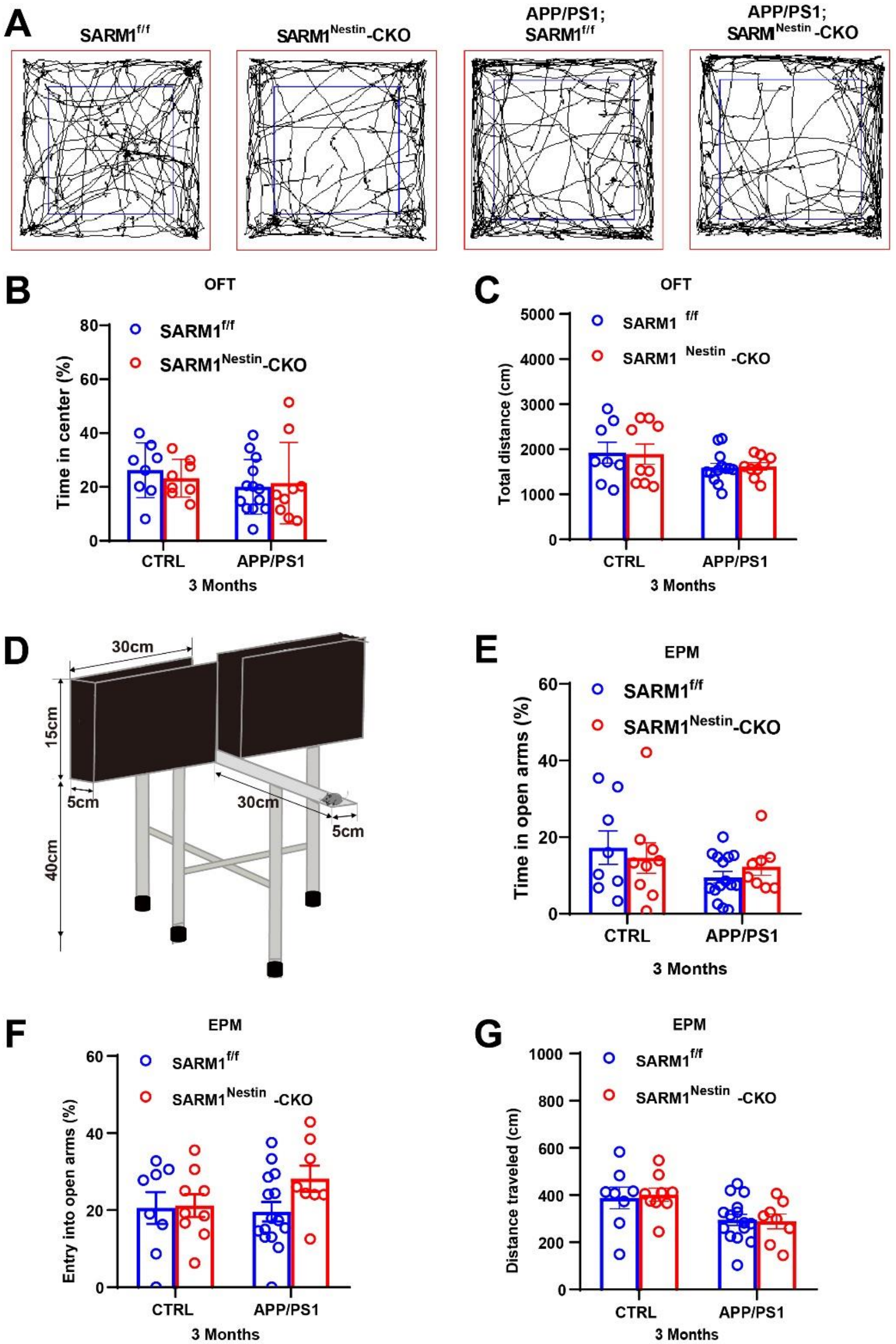

Supplementary Figure 3. SARM1 deletion in CNS did not exhibit anxiety-like behaviors in 3-month-old AD model mice. (A) Typical trajectory plots of 3-month-old SARM1$^{f/f}$ mice, SARM1^{Nestin}-CKO mice, APP/PS1;SARM1$^{f/f}$ mice and APP/PS1;SARM1^{Nestin}-CKO mice in the OFT. **(B)** Quantification of the percentage of time spent in the center of the open field in 3-month-old SARM1$^{f/f}$ mice (n = 8 mice), SARM1^{Nestin}-CKO mice (n = 9 mice), APP/PS1;SARM1$^{f/f}$ mice (n = 13 mice), APP/PS1;SARM1^{Nestin}-CKO mice (n = 9 mice). **(C)** Quantification of total distance traveled for locomotion in the center of the open field at 3-month-old SARM1$^{f/f}$ mice (n = 8 mice), SARM1^{Nestin}-CKO mice (n = 9 mice), APP/PS1;SARM1$^{f/f}$ mice (n = 13 mice), APP/PS1;SARM1^{Nestin}-CKO mice (n = 9 mice). **(D)** Schematic diagram of the EPM test. **(E)** Quantification of the percentage of time spent in the open arm of the EPM test at 3-month-old SARM1$^{f/f}$ mice (n = 8 mice), SARM1^{Nestin}-CKO mice (n = 9 mice), APP/PS1;SARM1$^{f/f}$ mice (n = 15 mice), APP/PS1;SARM1^{Nestin}-CKO mice (n = 8 mice). **(F)** Quantification of the percentage of time entering the open arm in the EPM test at 3-month-old SARM1$^{f/f}$ mice (n = 8 mice), SARM1^{Nestin}-CKO mice (n = 9 mice), APP/PS1;SARM1$^{f/f}$ mice (n = 15 mice), APP/PS1;SARM1^{Nestin}-CKO mice (n = 8 mice). **(G)** Total distance of exercise in EPM test at 3-month-old SARM1$^{f/f}$ mice (n = 8 mice), SARM1^{Nestin}-CKO mice (n = 9 mice), APP/PS1;SARM1$^{f/f}$ mice (n = 15 mice), APP/PS1;SARM1^{Nestin}-CKO mice (n = 8 mice). Data were mean ± SEM, one-way ANOVA with Tukey's post hoc analysis, compared with the control group.

© 2023. Miao X et al. Published online at http://www.aginganddisease.org/EN/10.14336/AD.2023.04516-1

SUPPLEMENTARY DATA

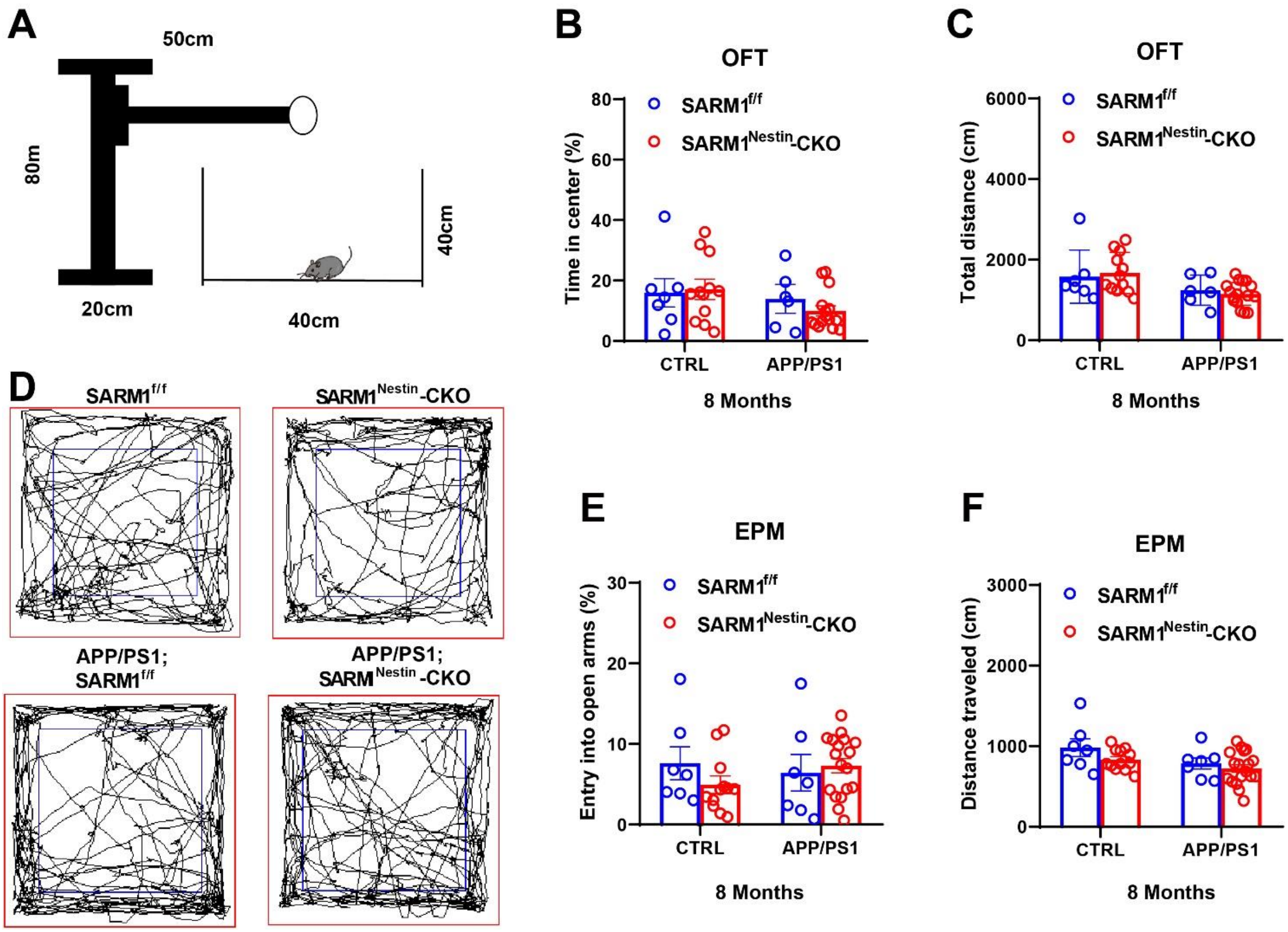

Supplementary Figure 4. SARM1 deletion in CNS did not exhibit anxiety-like behaviors in 8-month-old AD model mice. (A) Schematic diagram of the OFT. **(B)** Quantification of the percentage of time spent in the center of the open field in 8-month-old SARM1$^{f/f}$ mice (n = 7 mice), SARM1^{Nestin}-CKO mice (n = 12 mice), APP/PS1;SARM1$^{f/f}$ mice (n = 6 mice), APP/PS1;SARM1^{Nestin}-CKO mice (n = 16 mice). **(C)** Quantification of total distance traveled for locomotion in the center of the open field in 8-month-old SARM1$^{f/f}$ mice (n = 7 mice), SARM1^{Nestin}-CKO mice (n = 12 mice), APP/PS1;SARM1$^{f/f}$ mice (n = 6 mice), APP/PS1;SARM1^{Nestin}-CKO mice (n = 16 mice). **(D)** Typical trajectory plots of the OFT at 8-month-old in SARM1$^{f/f}$ mice, SARM1^{Nestin}-CKO mice, APP/PS1;SARM1$^{f/f}$ mice, APP/PS1;SARM1^{Nestin}-CKO mice. **(E)** Quantification of the percentage of time spent in the open arm of the EPM test at 8-month-old SARM1$^{f/f}$ mice (n = 7 mice), SARM1^{Nestin}-CKO mice (n = 12 mice), APP/PS1;SARM1$^{f/f}$ mice (n = 7 mice), APP/PS1;SARM1^{Nestin}-CKO mice (n = 18 mice). **(F)** Quantification of total distance traveled for exercise in EPM test at 8-month-old in SARM1$^{f/f}$ mice (n = 7 mice), SARM1^{Nestin}-CKO mice (n = 12 mice), APP/PS1;SARM1$^{f/f}$ mice (n = 7 mice), APP/PS1;SARM1^{Nestin}-CKO mice (n = 18 mice). Data were mean ± SEM, one-way ANOVA with Tukey's post hoc analysis, compared with the control group.

© 2023. Miao X et al. Published online at http://www.aginganddisease.org/EN/10.14336/AD.2023.04516-1

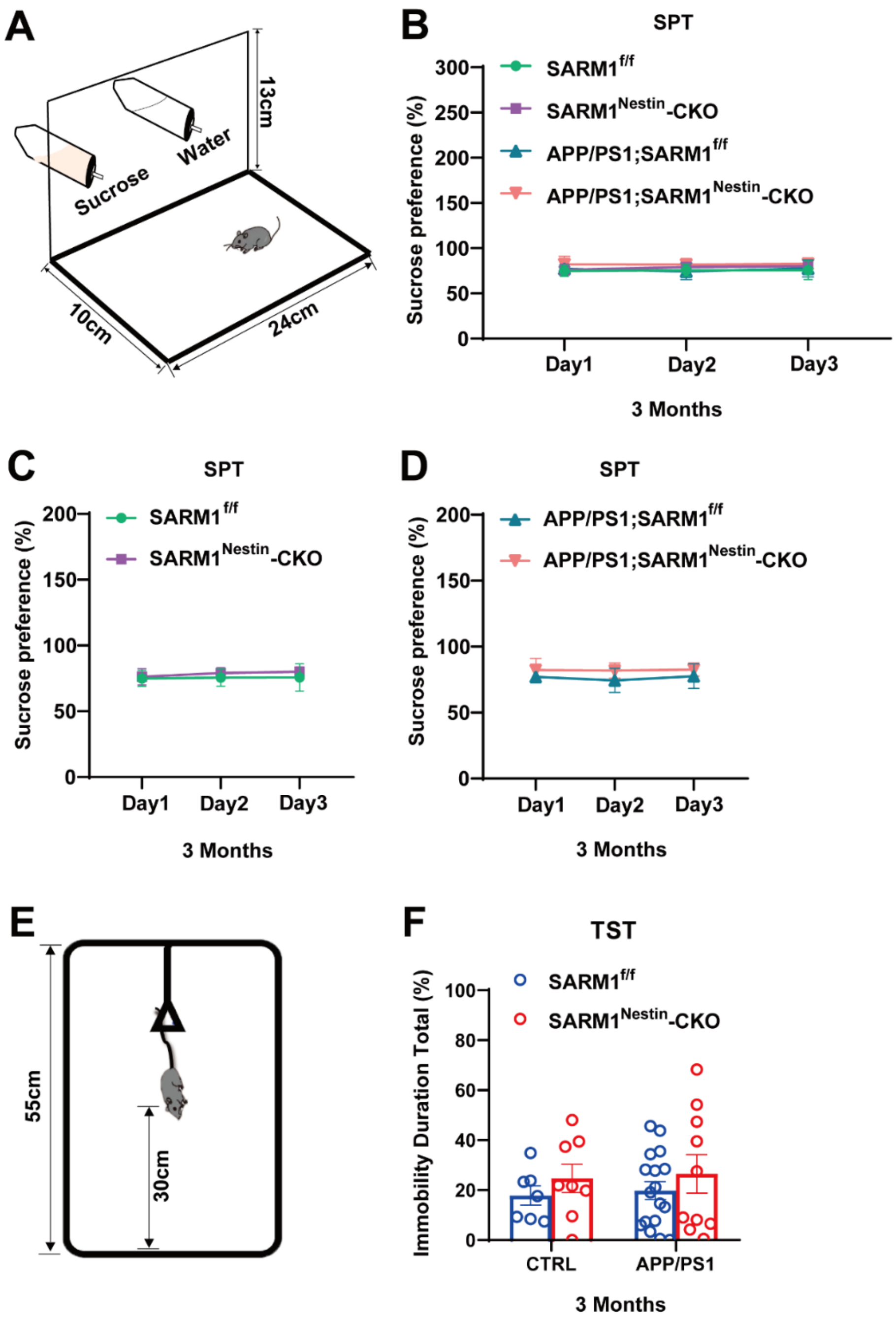

Supplementary Figure 5. SARM1 deletion in CNS did not exhibit depression-like behaviors in 3-month-old AD model mice. (A) Schematic diagram of the SPT. **(B)** Quantification of sucrose preference index in SPT at 3-month-old SARM1$^{f/f}$ mice (n = 7 mice), SARM1^{Nestin}-CKO mice (n = 8 mice), APP/PS1;SARM1$^{f/f}$ mice (n = 17 mice), APP/PS1;SARM1^{Nestin}-CKO mice (n = 10 mice). **(C)** Quantification of sucrose preference index in SPT in 3-month-old SARM1$^{f/f}$ mice (n = 7 mice), SARM1^{Nestin}-CKO mice (n = 8 mice). **(D)** Quantification of sucrose preference index in SPT at 3-month-old APP/PS1;SARM1$^{f/f}$ mice (n = 17 mice), APP/PS1;SARM1^{Nestin}-CKO mice (n = 10 mice). **(E)** Schematic diagram of the TST. **(F)** Quantification of the percentage of immobility time in the TST at 3-month-old SARM1$^{f/f}$ mice (n = 7 mice), SARM1^{Nestin}-CKO mice (n = 8 mice), APP/PS1;SARM1$^{f/f}$ mice (n = 17 mice), APP/PS1;SARM1^{Nestin}-CKO mice (n = 10 mice). Data were mean ± SEM, one-way ANOVA with Tukey's post hoc analysis, compared with the control group.

© 2023. Miao X et al. Published online at http://www.aginganddisease.org/EN/10.14336/AD.2023.04516-1

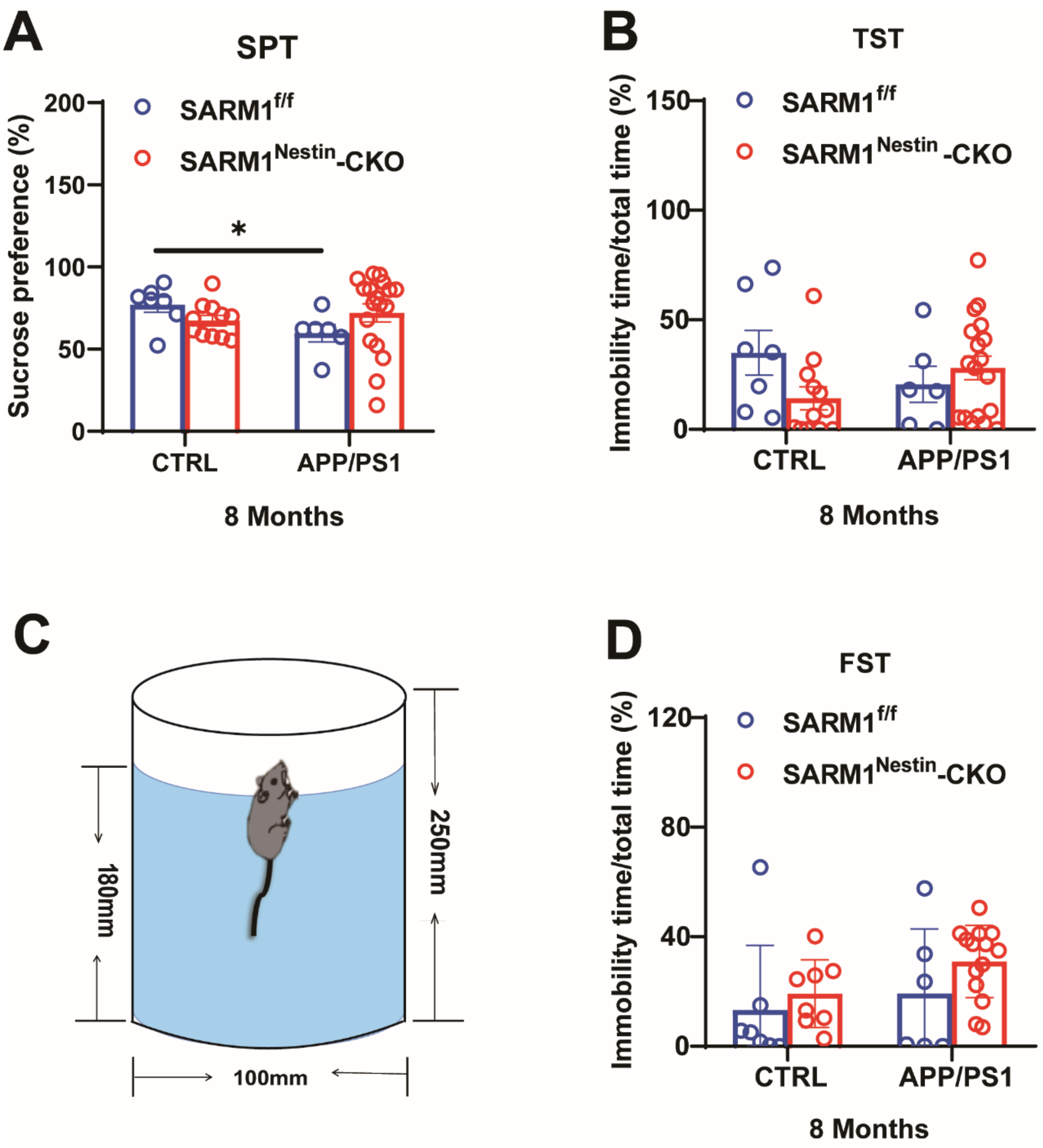

Supplementary Figure 6. SARM1 deletion in CNS did not exhibit depression-like behaviors in 8-month-old AD model mice. (A) Quantification of sucrose preference index in SPT at 8-month-old SARM1$^{f/f}$ mice (n = 7 mice), SARM1^{Nestin}-CKO mice (n = 11 mice), APP/PS1;SARM1$^{f/f}$ mice (n = 6 mice), APP/PS1;SARM1^{Nestin}-CKO mice (n = 18 mice). **(B)** Quantification of the percentage of immobility time in the TST at 8-month-old SARM1$^{f/f}$ mice (n = 7 mice), SARM1^{Nestin}-CKO mice (n = 11 mice), APP/PS1;SARM1$^{f/f}$ mice (n = 6 mice), APP/PS1;SARM1^{Nestin}-CKO mice (n = 18 mice). **(C)** Schematic diagram of FST. **(D)** Quantification of percentage of immobility time in the FST at 8-month-old SARM1$^{f/f}$ mice (n = 7 mice), SARM1^{Nestin}-CKO mice (n = 8 mice), APP/PS1;SARM1$^{f/f}$ mice (n = 6 mice), APP/PS1;SARM1^{Nestin}-CKO mice (n = 14 mice). Data were mean ± SEM, $^{*}P < 0.05$, one-way ANOVA with Tukey's post hoc analysis, compared with the control group.

© 2023. Miao X et al. Published online at http://www.aginganddisease.org/EN/10.14336/AD.2023.04516-1

SUPPLEMENTARY DATA

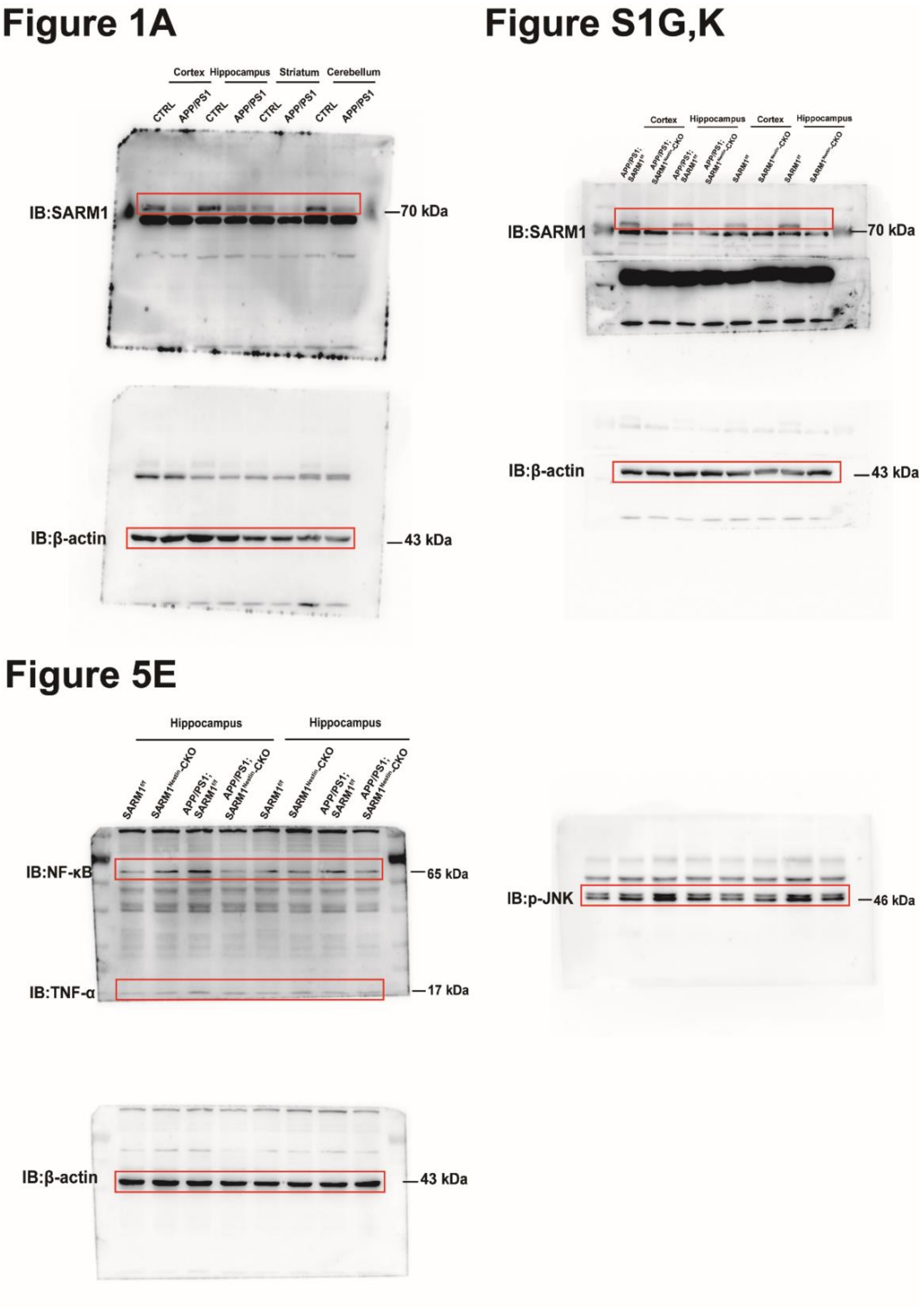

Supplementary Figure 7. The raw data of Western blot of the entire incubated film. (Fig. 1A) Western blot detected the expression of SARM1 in the cortex, hippocampus, striatum and cerebellum of 9-month-old WT mice and APP/PS1 mice. **(Supplementary Fig. 1G, K)** Western blot detected the expression of SARM1 in the hippocampus and cortex of 9-month-old SARM1$^{f/f}$ mice, SARM1^{Nestin}-CKO mice, APP/PS1;SARM1$^{f/f}$ mice and APP/PS1;SARM^{1Nestin}-CKO mice. **(Fig. 5E)** Western blot detected the expression levels of TNF-α, NF-κB and p-JNK in the hippocampus of 9-month-old SARM1$^{f/f}$ mice, SARM1^{Nestin}-CKO mice, APP/PS1;SARM1$^{f/f}$ mice and APP/PS1;SARM1^{Nestin}-CKO mice.

© 2023. Miao X et al. Published online at http://www.aginganddisease.org/EN/10.14336/AD.2023.04516-1
